# Supplementary material for: STX4 Is Indispensable for Mitochondrial Homeostasis in Skeletal Muscle
Source: J Cachexia Sarcopenia Muscle. 2025 Nov 10;16(6):e70113. doi: 10.1002/jcsm.70113 (PMC12602274; doi:10.1002/jcsm.70113)
Supplement: Supplementary file 1 — Data S1: Supporting Information. [file JCSM-16-e70113-s003.pdf]

Raw Blots File

STX4 is indispensable for mitochondrial homeostasis in skeletal muscle

Joseph M. Hoolachan[1], Rekha Balakrishnan[1], Erika M. McCown[1], Karla E. Merz[1,2], Chunxue Zhou[1,3], Elizabeth Bloom-Saldana[1,4], Patrick T. Fueger[1,4], Angelica Hamilton[1,5], Tali Kiperman[6], Ke Ma[6], Eunjin Oh[1], Lei Jiang[7], Patrick Pirrotte[7,8], Orian Shirihai[9] & Debbie C. Thurmond[1].

1. Department of Molecular and Cellular Endocrinology, Arthur Riggs Diabetes and Metabolism Research Institute, City of Hope Beckman Research Institute, 1500 E. Duarte Road, Duarte, CA 91010, USA.
2. Amgen Inc, Thousand Oaks, CA, USA.
3. Sanegene Bio Inc. Suzhou, China.
4. Comprehensive Metabolic Phenotyping Core, Beckman Research Institute, 1500 E. Duarte Road, Duarte, CA, 91010, USA.
5. Clinical Research Division MC, City of Hope Beckman Research Institute, 1500 E. Duarte Road, Duarte, CA 91010, USA.
6. Department of Diabetes Complications and Metabolism, Arthur Riggs Diabetes and Metabolism Research Institute, City of Hope Beckman Research Institute, 1500 E. Duarte Road, Duarte, CA 91010, USA.
7. Integrated Mass Spectrometry Shared Resource, City of Hope Beckman Research Institute, 1500 E. Duarte Road, Duarte, CA 91010 USA.
8. Early Detection & Prevention Division, Translational Genomics Research Institute, 445 N. 5th Str, Phoenix, AZ, 85004, USA.
9. Department of Medicine, University of California Los Angeles, 100 Medical Plaza, Los Angeles, CA, 90095, USA.

Fig. 1a

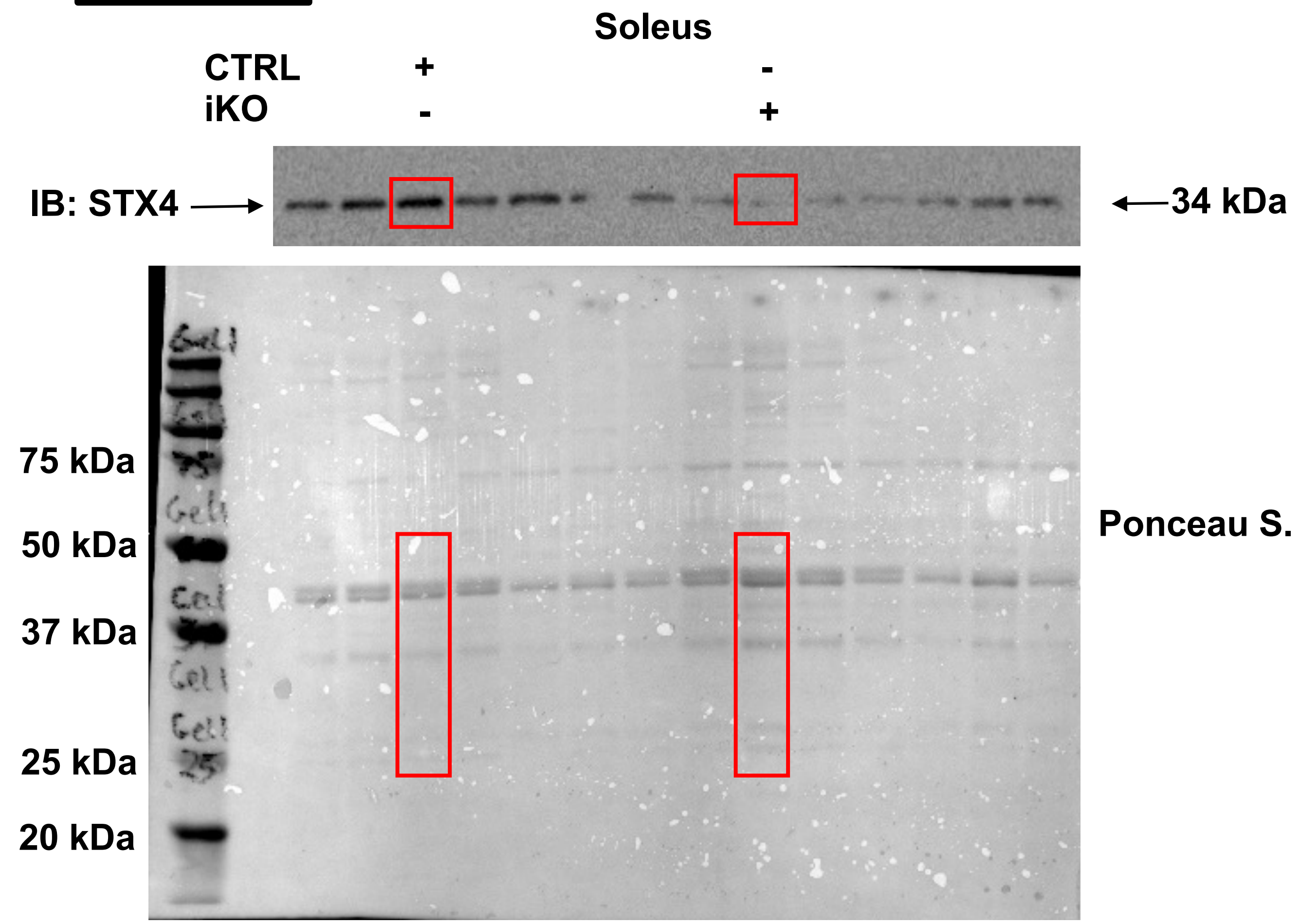

Soleus
CTRL + -
iKO - +
IB: STX4
34 kDa
75 kDa
50 kDa
37 kDa
25 kDa
20 kDa
Ponceau S.

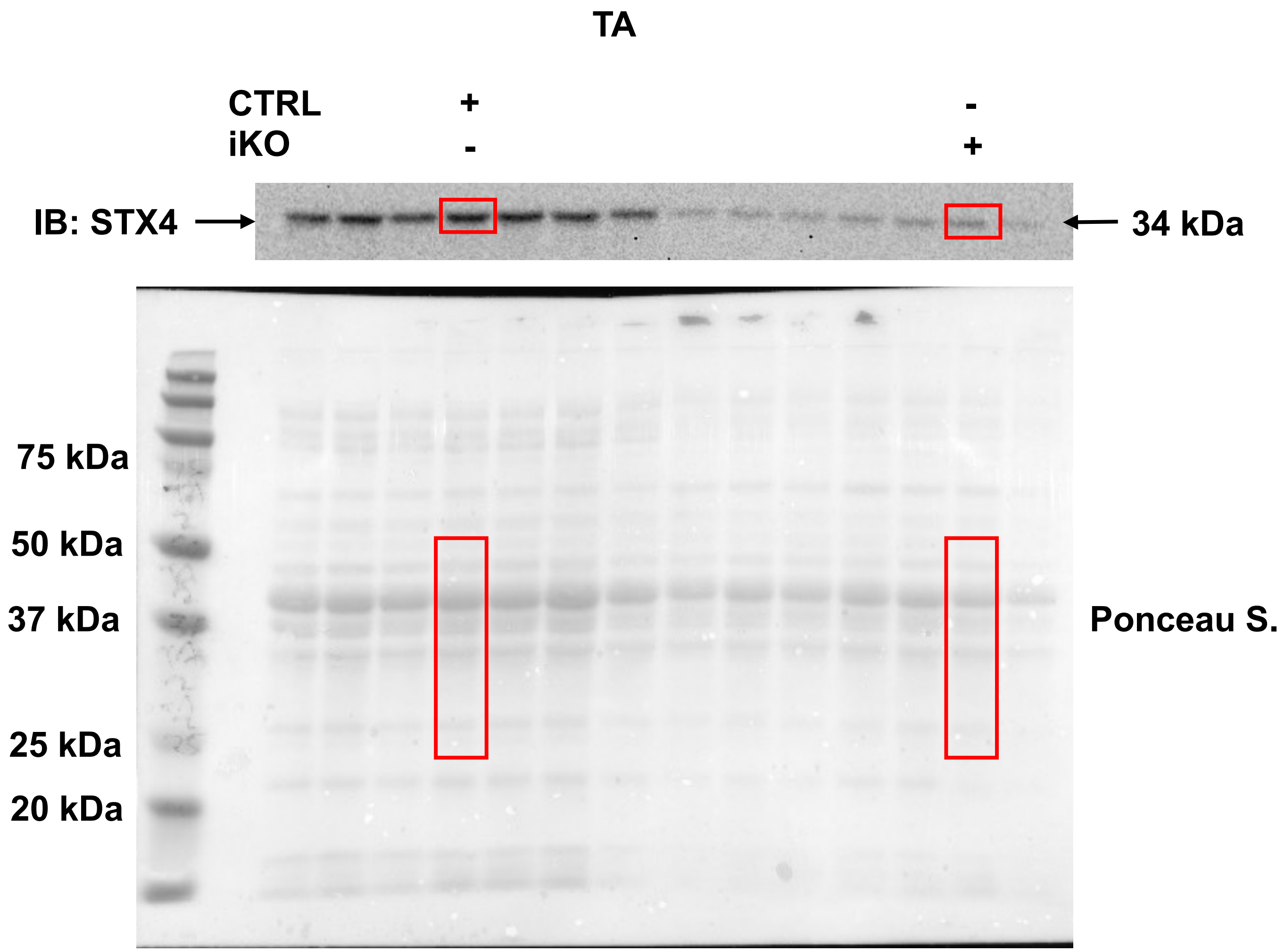

TA
CTRL + -
iKO - +
IB: STX4
34 kDa
75 kDa
50 kDa
37 kDa
25 kDa
20 kDa
Ponceau S.

Fig. 1a

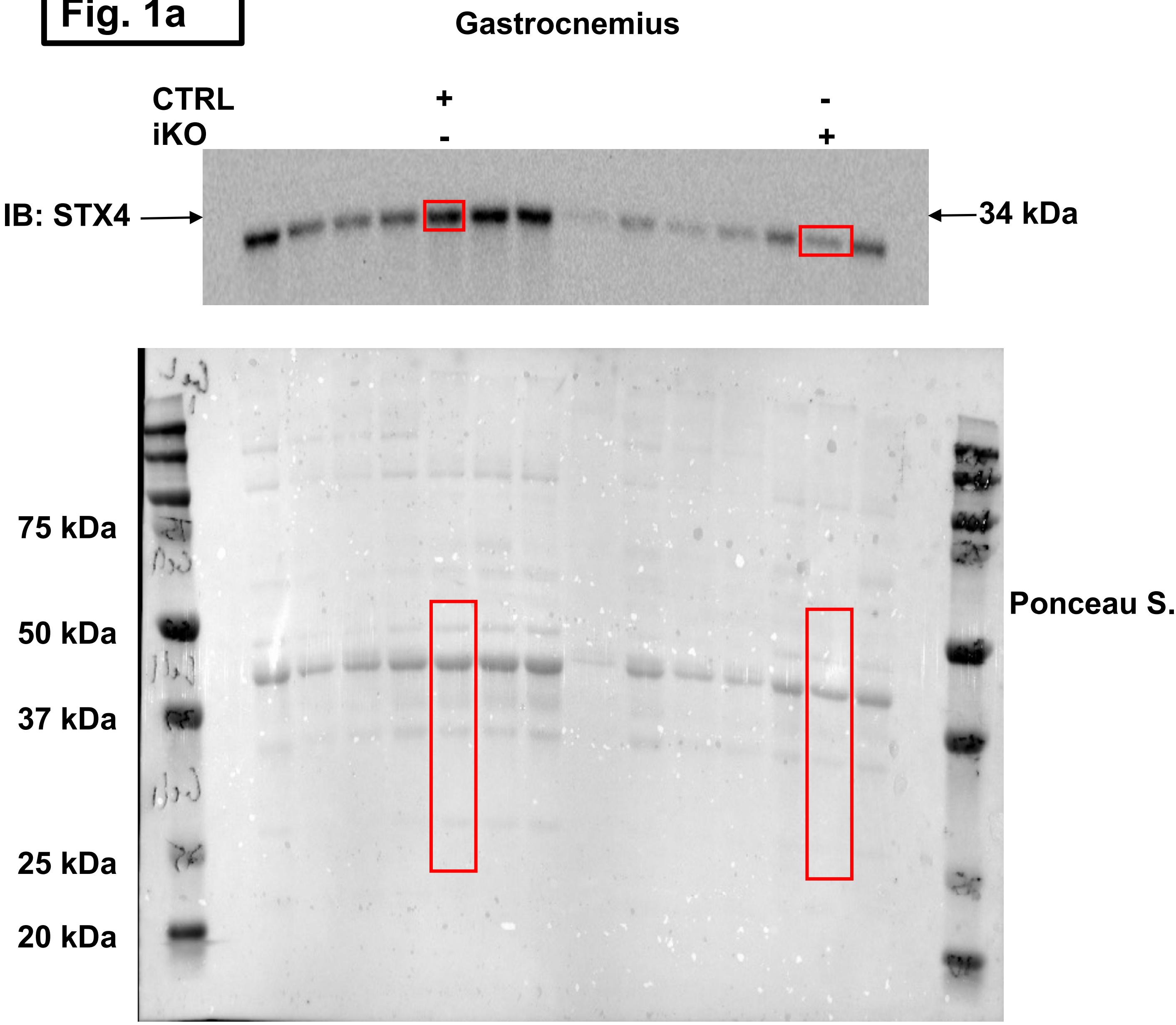

Gastrocnemius
CTRL
iKO
+
-
-
+
IB: STX4
34 kDa
75 kDa
50 kDa
37 kDa
25 kDa
20 kDa
Ponceau S.

Fig. 1b

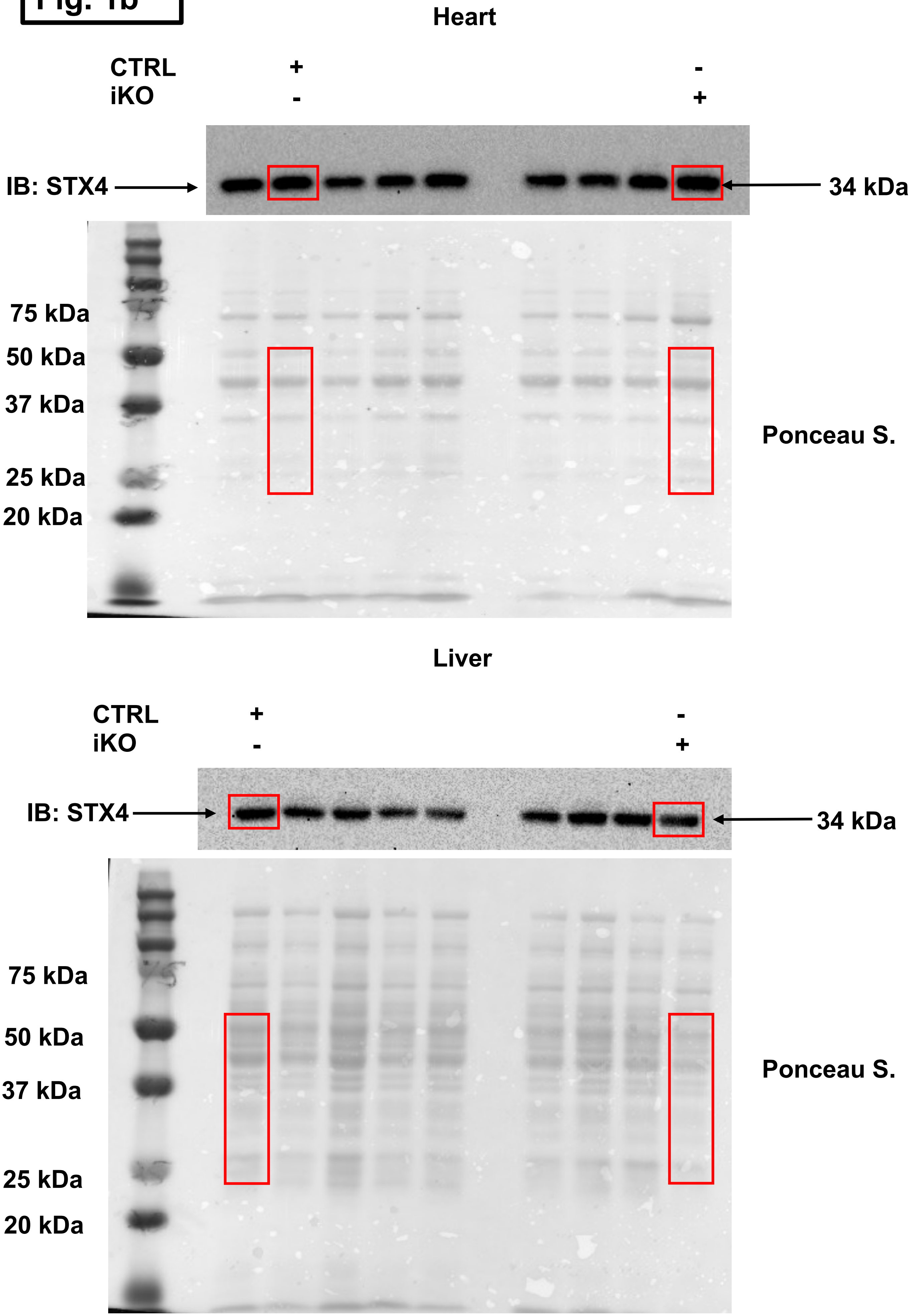
Heart
CTRL + -
iKO - +
IB: STX4
34 kDa
75 kDa
50 kDa
37 kDa
25 kDa
20 kDa
Ponceau S.
Liver
CTRL + -
iKO - +
IB: STX4
34 kDa
75 kDa
50 kDa
37 kDa
25 kDa
20 kDa
Ponceau S.

Fig. 3c

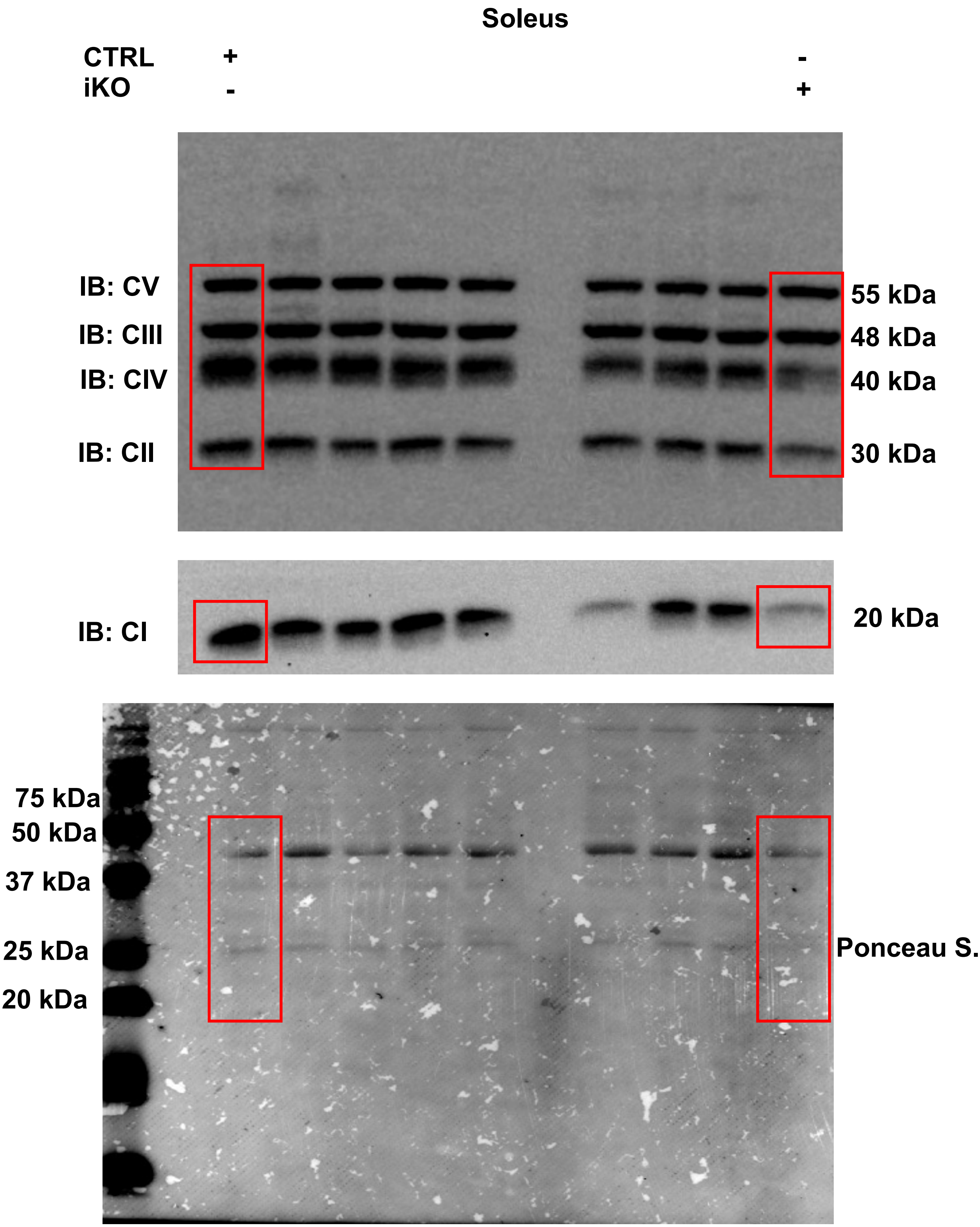

Soleus
CTRL
+
-
iKO
-
+
IB: CV
IB: CIII
IB: CIV
IB: CII
55 kDa
48 kDa
40 kDa
30 kDa
IB: CI
20 kDa
75 kDa
50 kDa
37 kDa
25 kDa
20 kDa
Ponceau S.

Fig. 3d

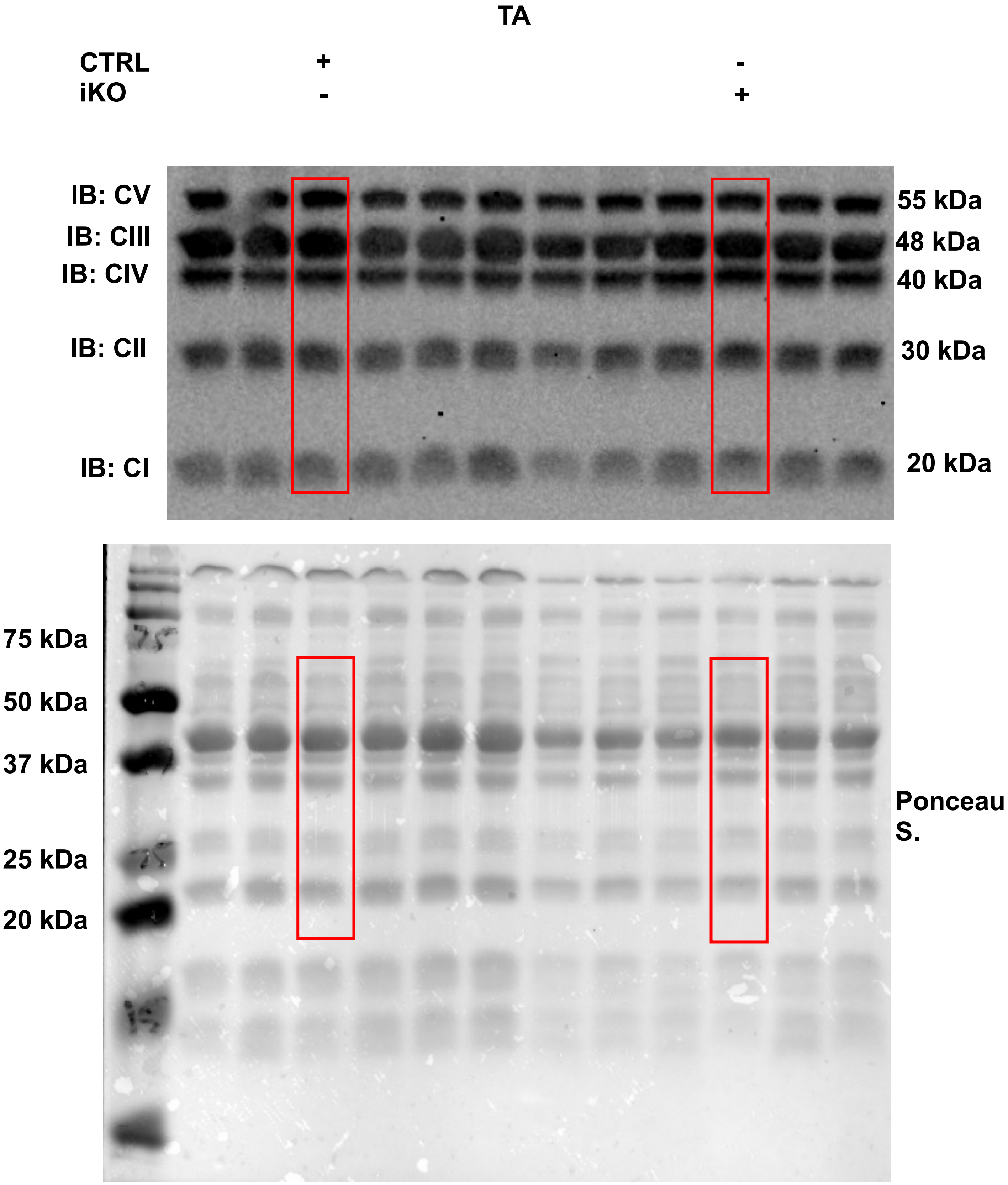
TA
CTRL
+
-
iKO
-
+
IB: CV
IB: CIII
IB: CIV
IB: CII
IB: CI
55 kDa
48 kDa
40 kDa
30 kDa
20 kDa
75 kDa
50 kDa
37 kDa
25 kDa
20 kDa
Ponceau S.

Fig. 4b

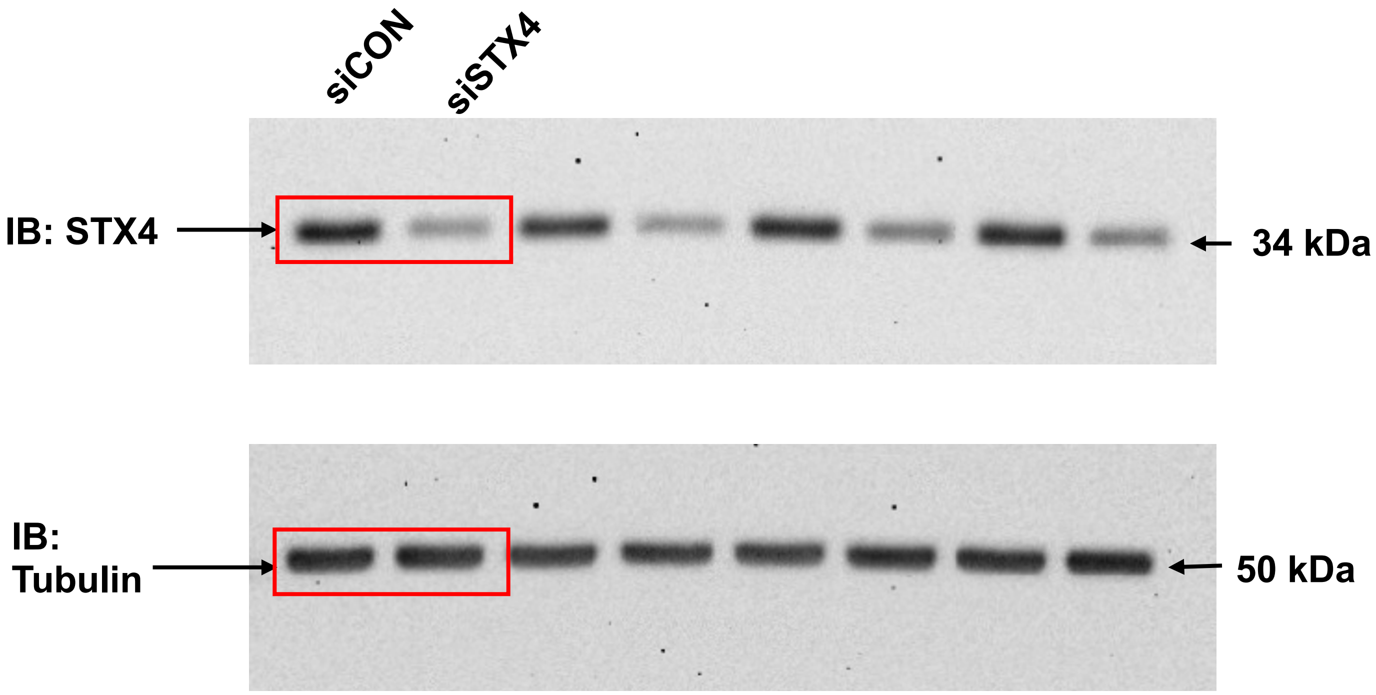

Fig. 4c

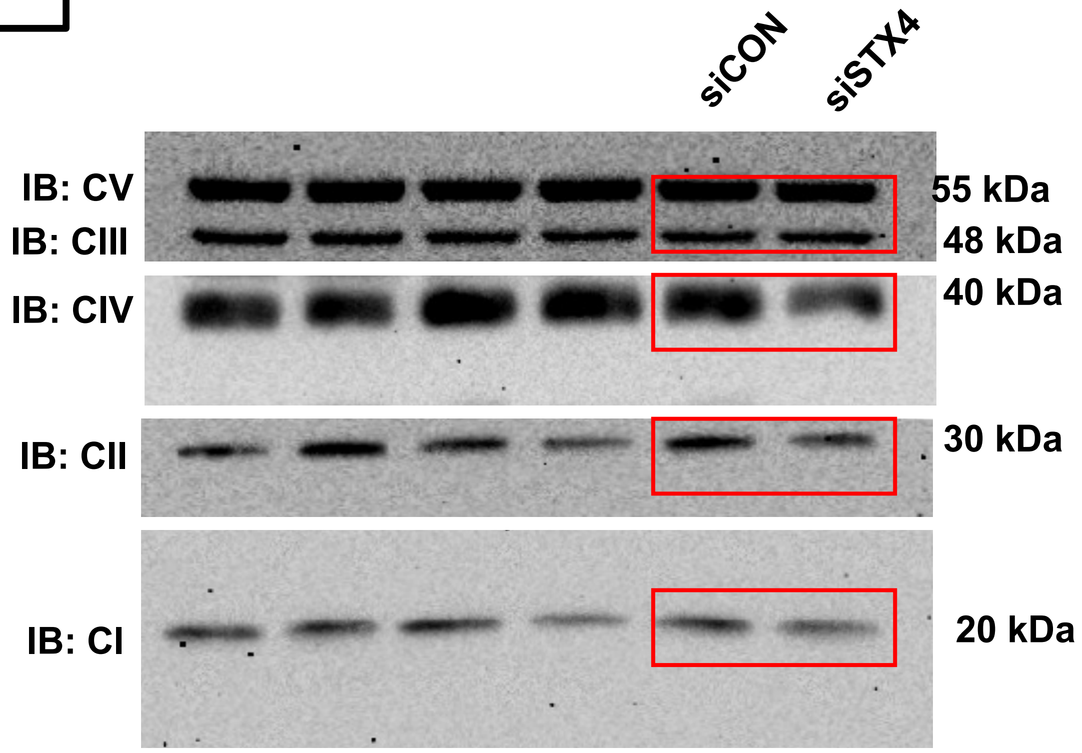
siCON
siSTX4
IB: CV
IB: CIII
IB: CIV
IB: CII
IB: CI
55 kDa
48 kDa
40 kDa
30 kDa
20 kDa

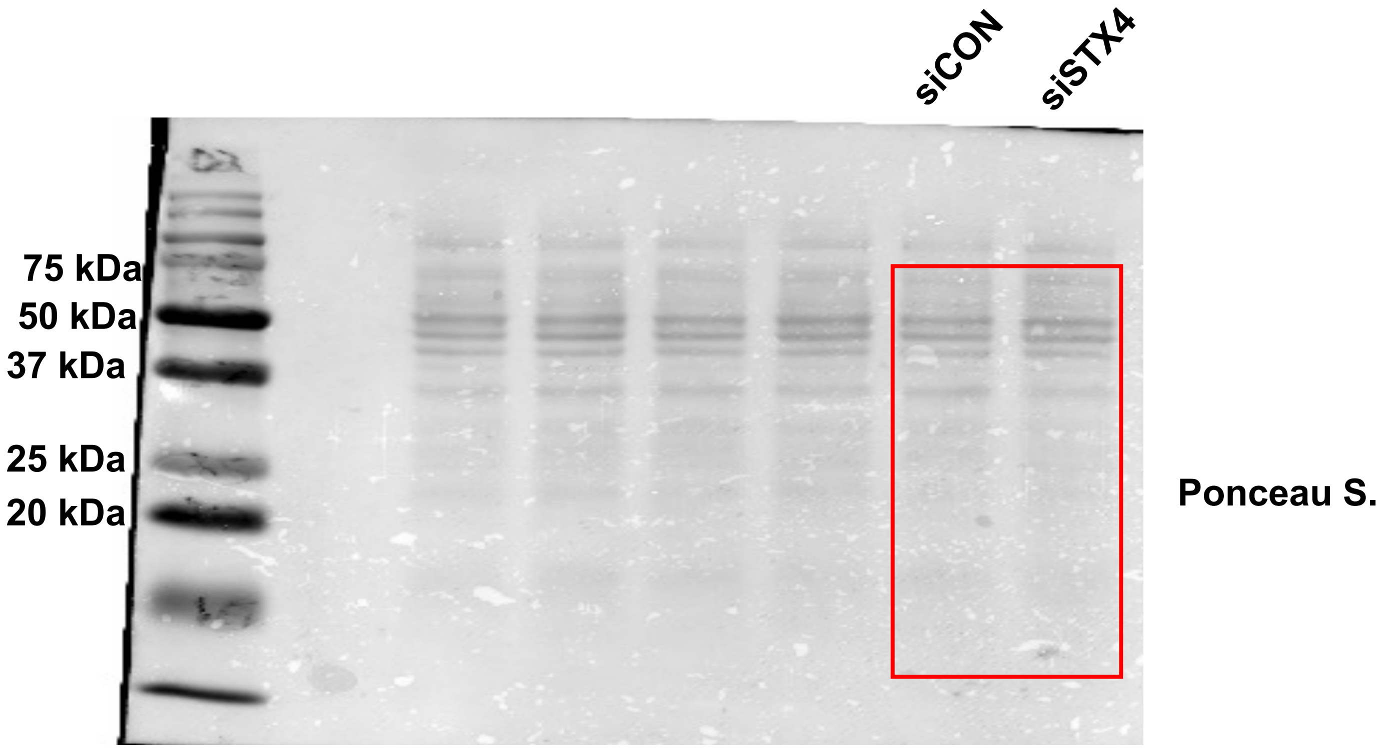
siCON
siSTX4
75 kDa
50 kDa
37 kDa
25 kDa
20 kDa
Ponceau S.

Fig. 5e

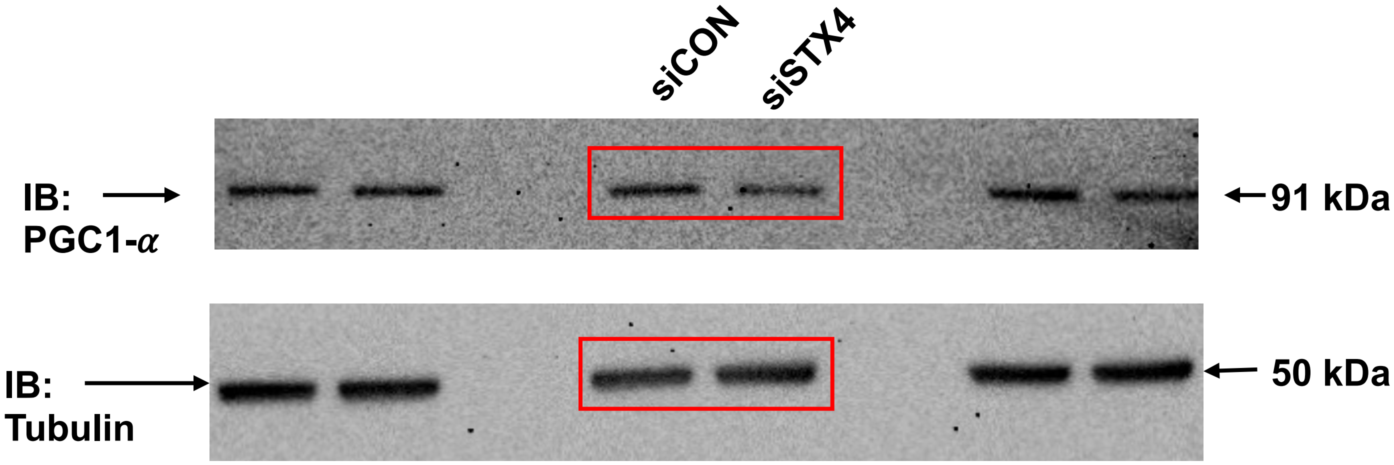

Fig. 5f

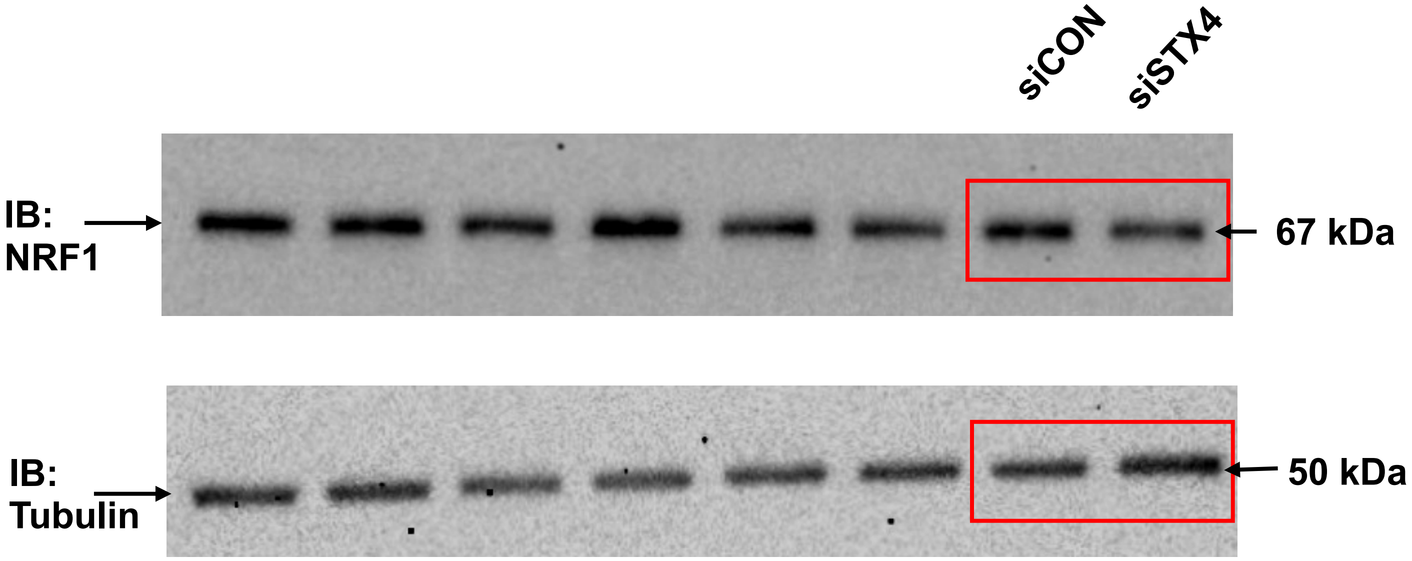

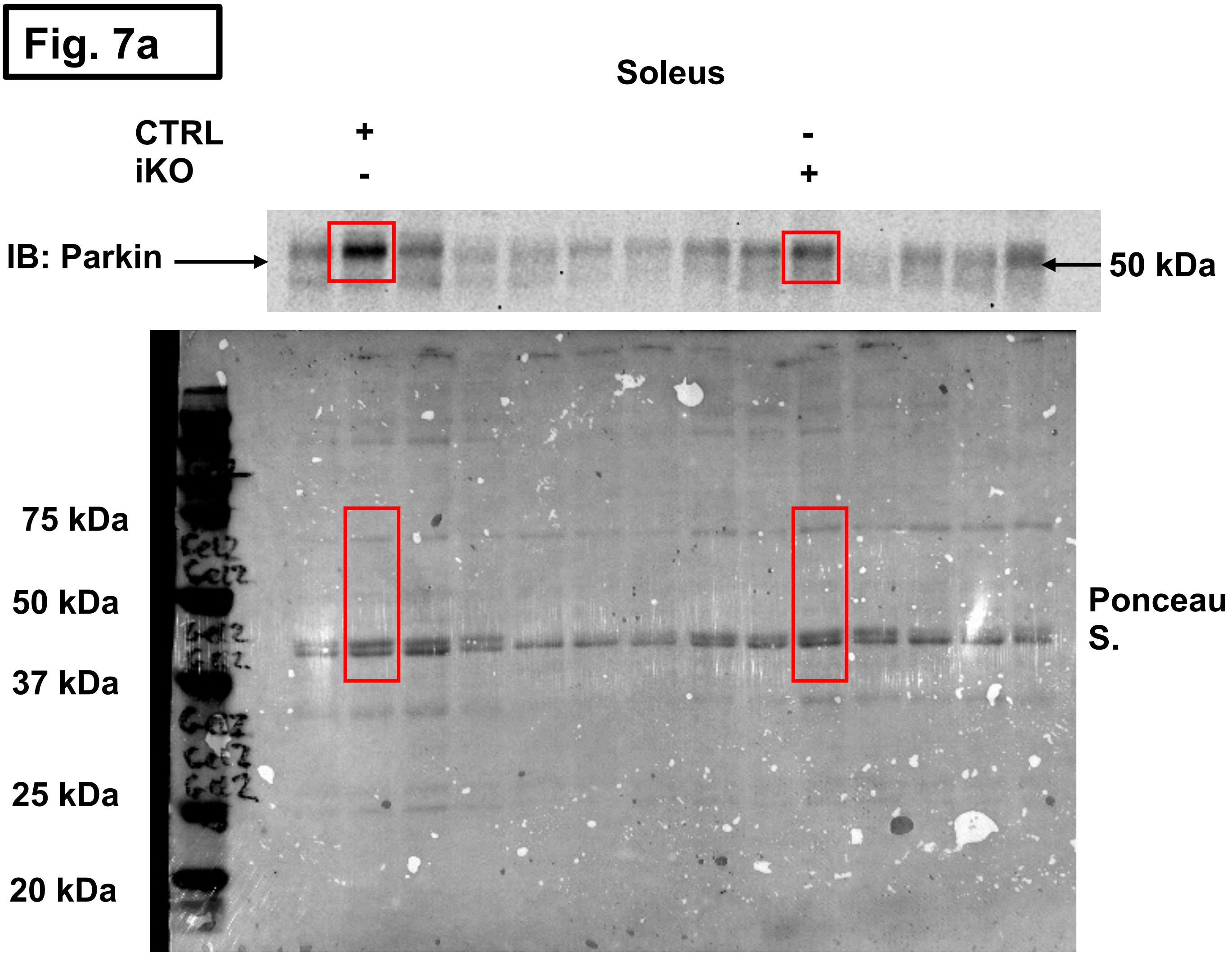
Fig. 7a
Soleus
CTRL + -
iKO - +
IB: Parkin
50 kDa
75 kDa
50 kDa
37 kDa
25 kDa
20 kDa
Ponceau S.

Fig. 7a

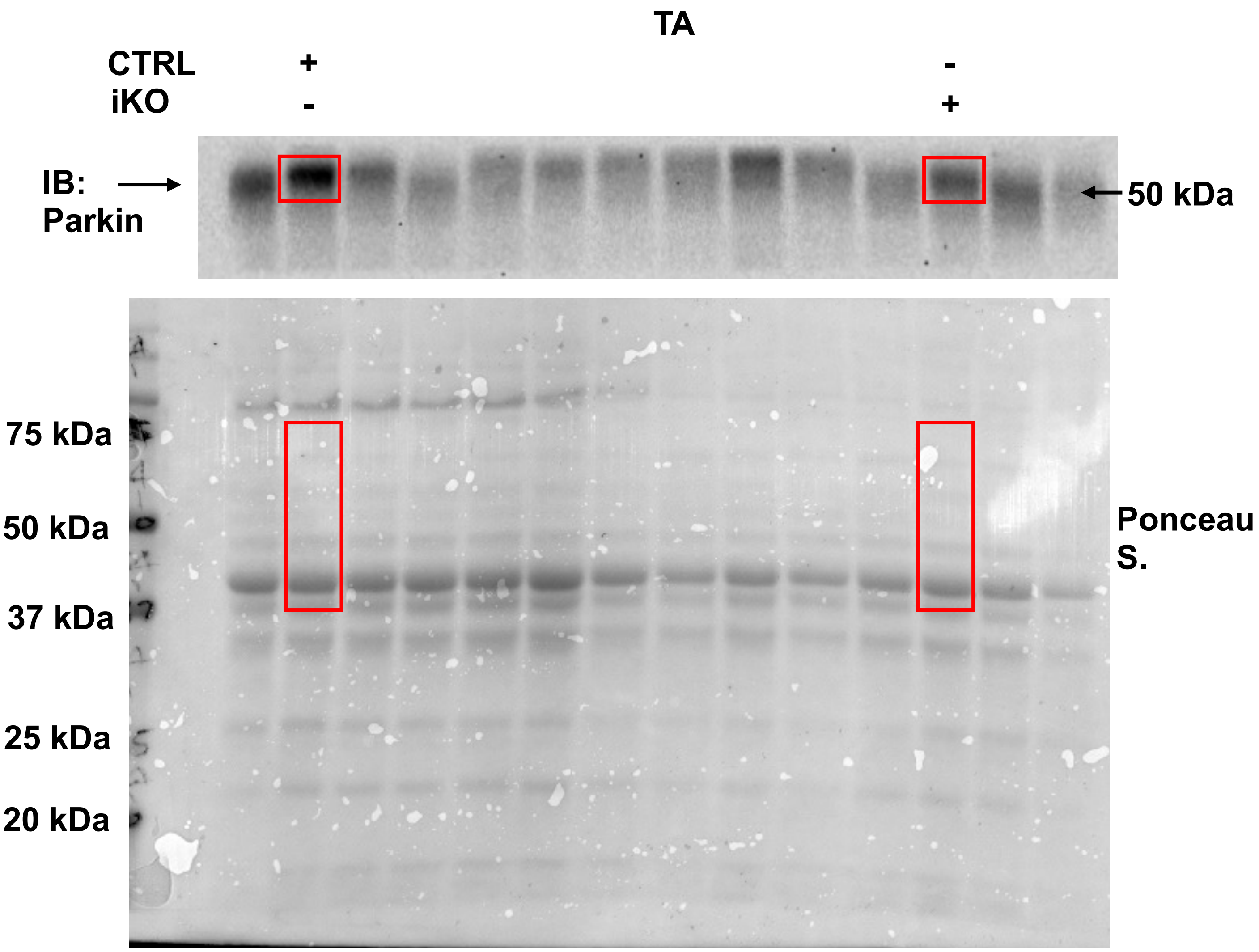
TA
CTRL + -
iKO - +
IB: Parkin
50 kDa
75 kDa
50 kDa
37 kDa
25 kDa
20 kDa
Ponceau S.

Fig. 7b

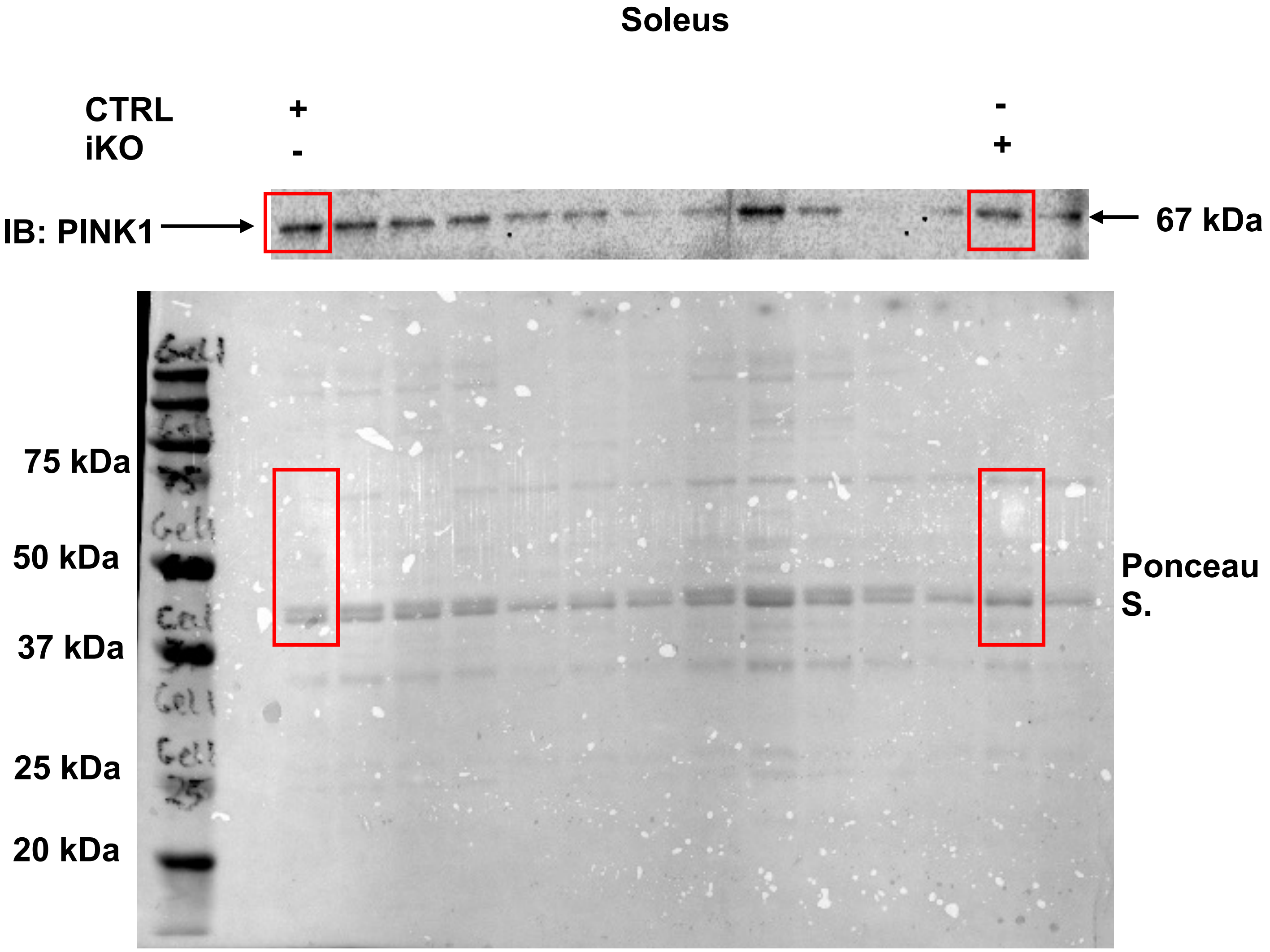

Soleus
CTRL + -
iKO - +
IB: PINK1
67 kDa
75 kDa
50 kDa
37 kDa
25 kDa
20 kDa
Ponceau S.

Fig. 7b

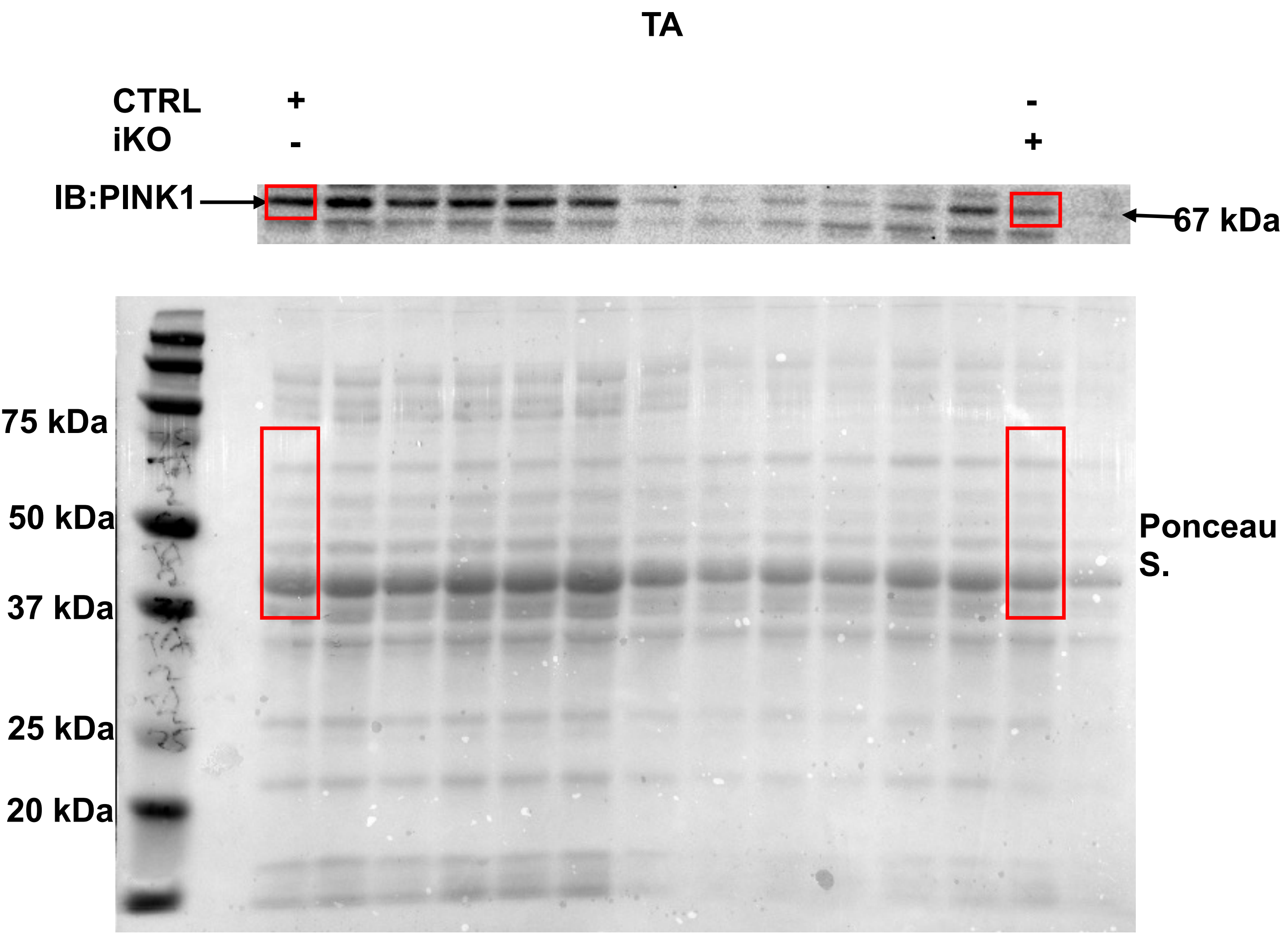
TA
CTRL
+
-
iKO
-
+
IB:PINK1
67 kDa
75 kDa
50 kDa
37 kDa
25 kDa
20 kDa
Ponceau S.

Fig. 7c

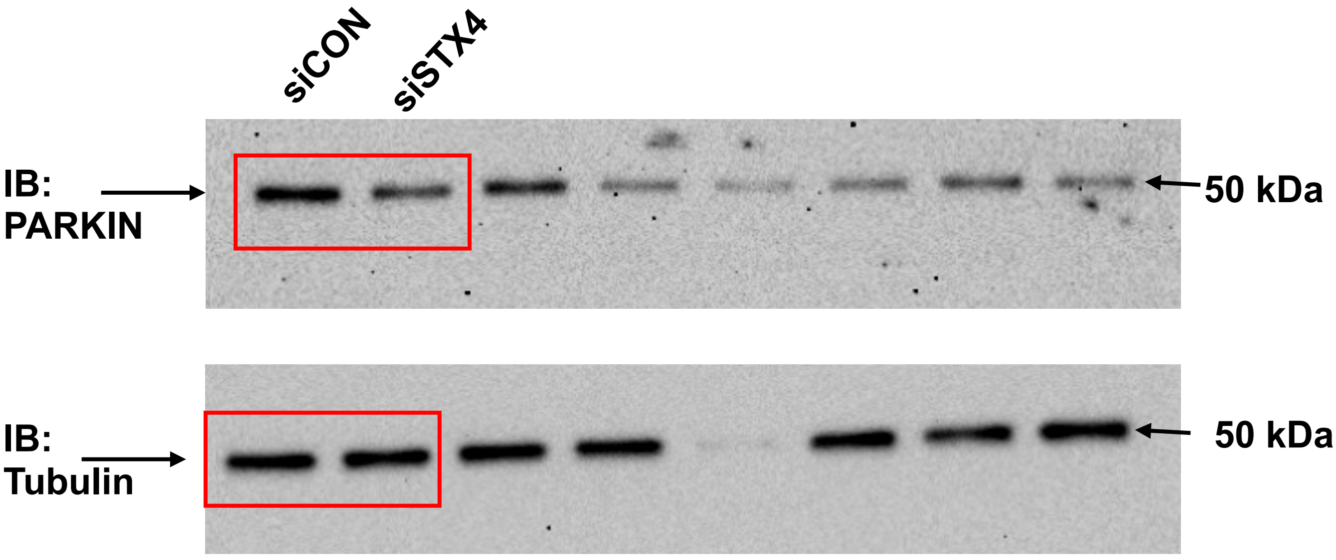

Fig. 7d

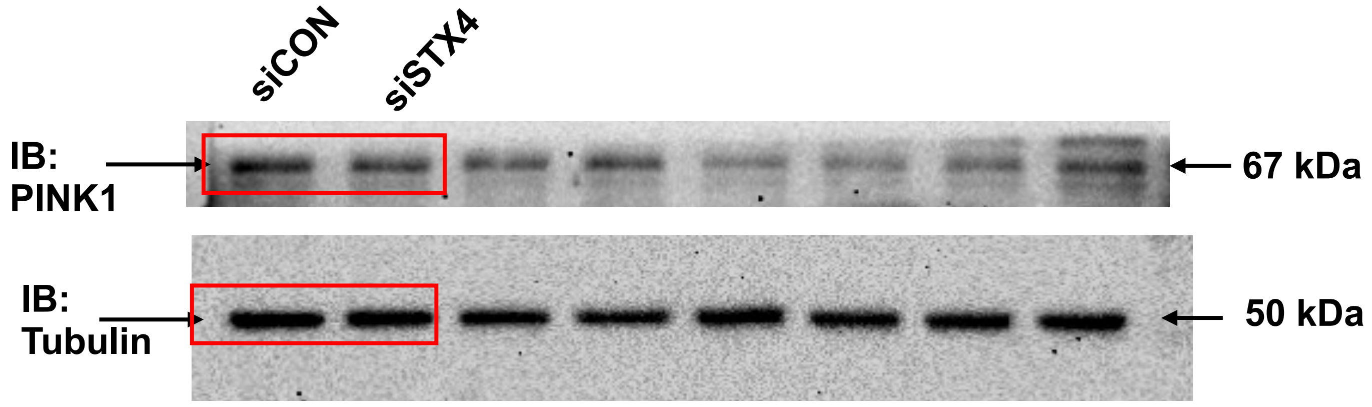

Fig. S2a

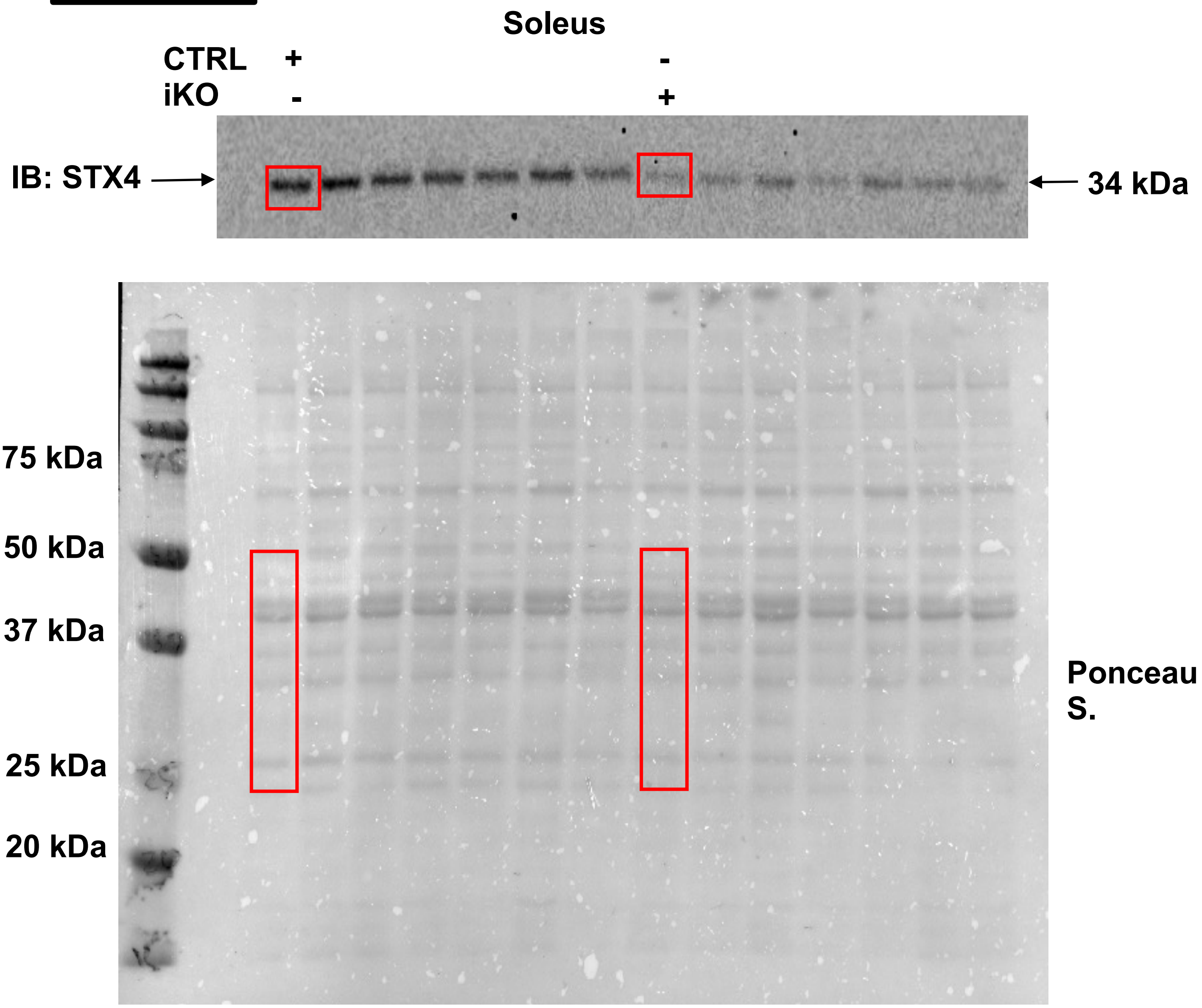
Soleus
CTRL + -
iKO - +
IB: STX4
34 kDa
75 kDa
50 kDa
37 kDa
25 kDa
20 kDa
Ponceau S.

Fig. S2b

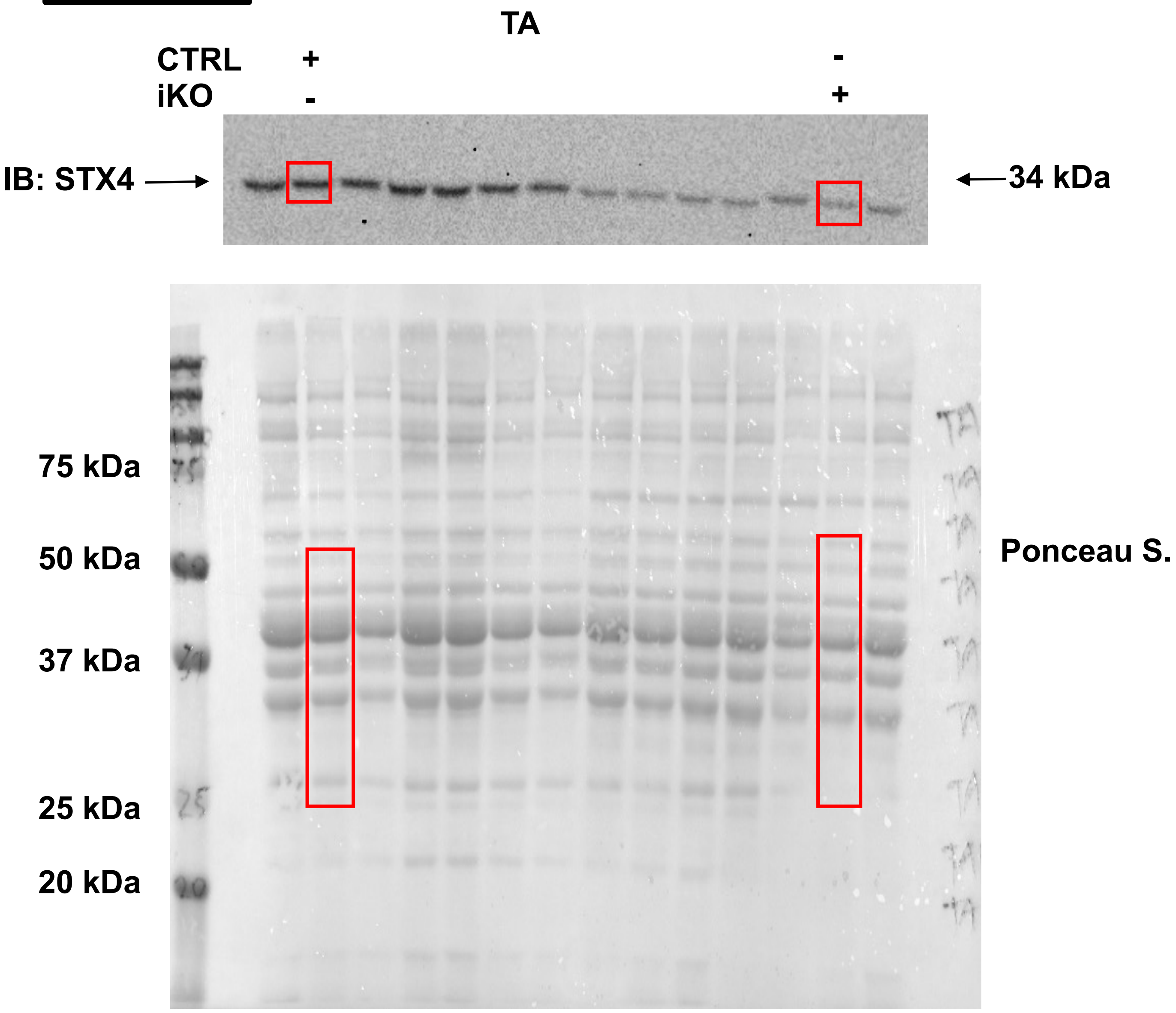
TA
CTRL + -
iKO - +
IB: STX4
34 kDa
75 kDa
50 kDa
37 kDa
25 kDa
20 kDa
Ponceau S.

Fig. S4c

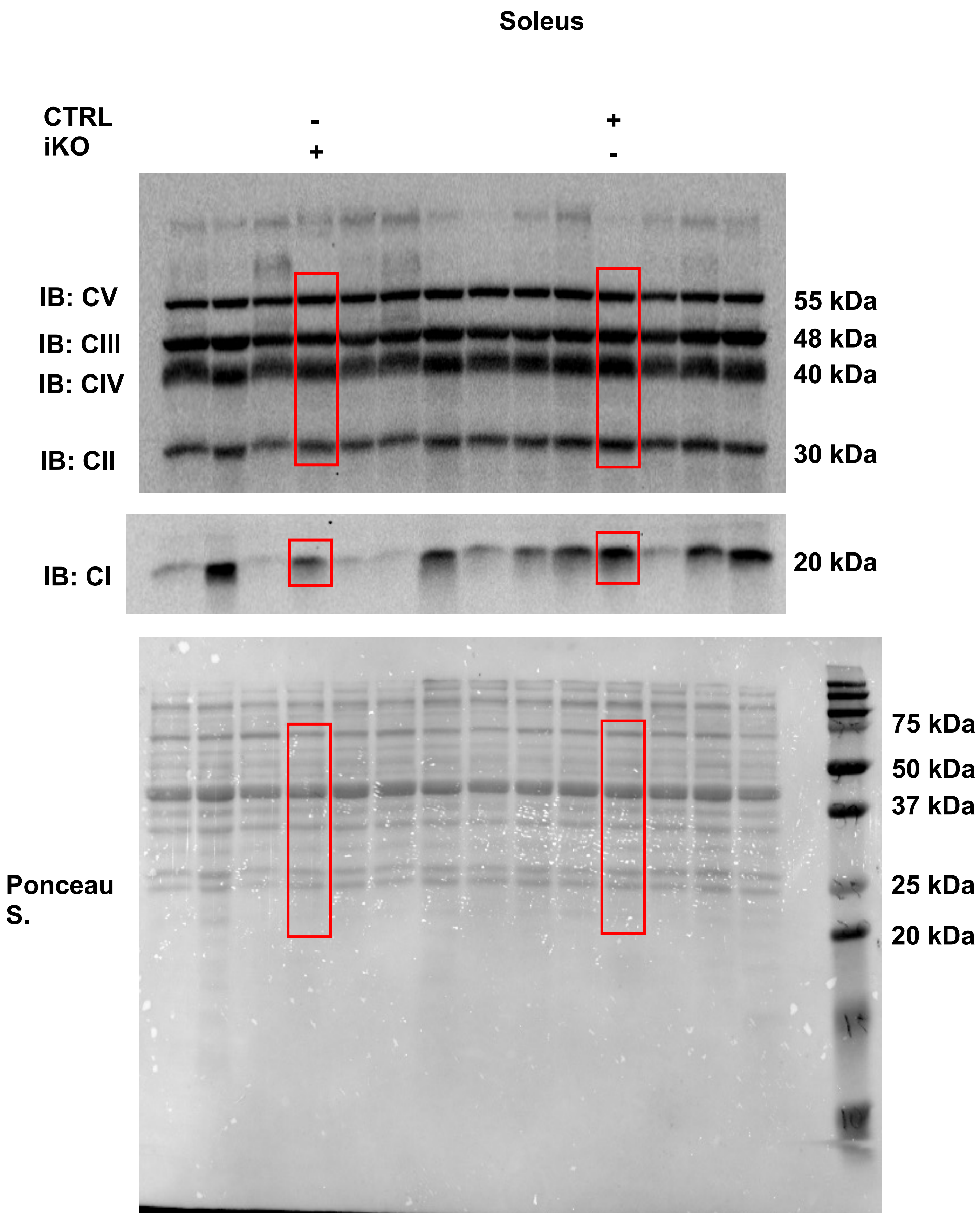
Soleus
CTRL
iKO
-
+
+
-
IB: CV
IB: CIII
IB: CIV
IB: CII
55 kDa
48 kDa
40 kDa
30 kDa
IB: CI
20 kDa
Ponceau S.
75 kDa
50 kDa
37 kDa
25 kDa
20 kDa

Fig. S4d

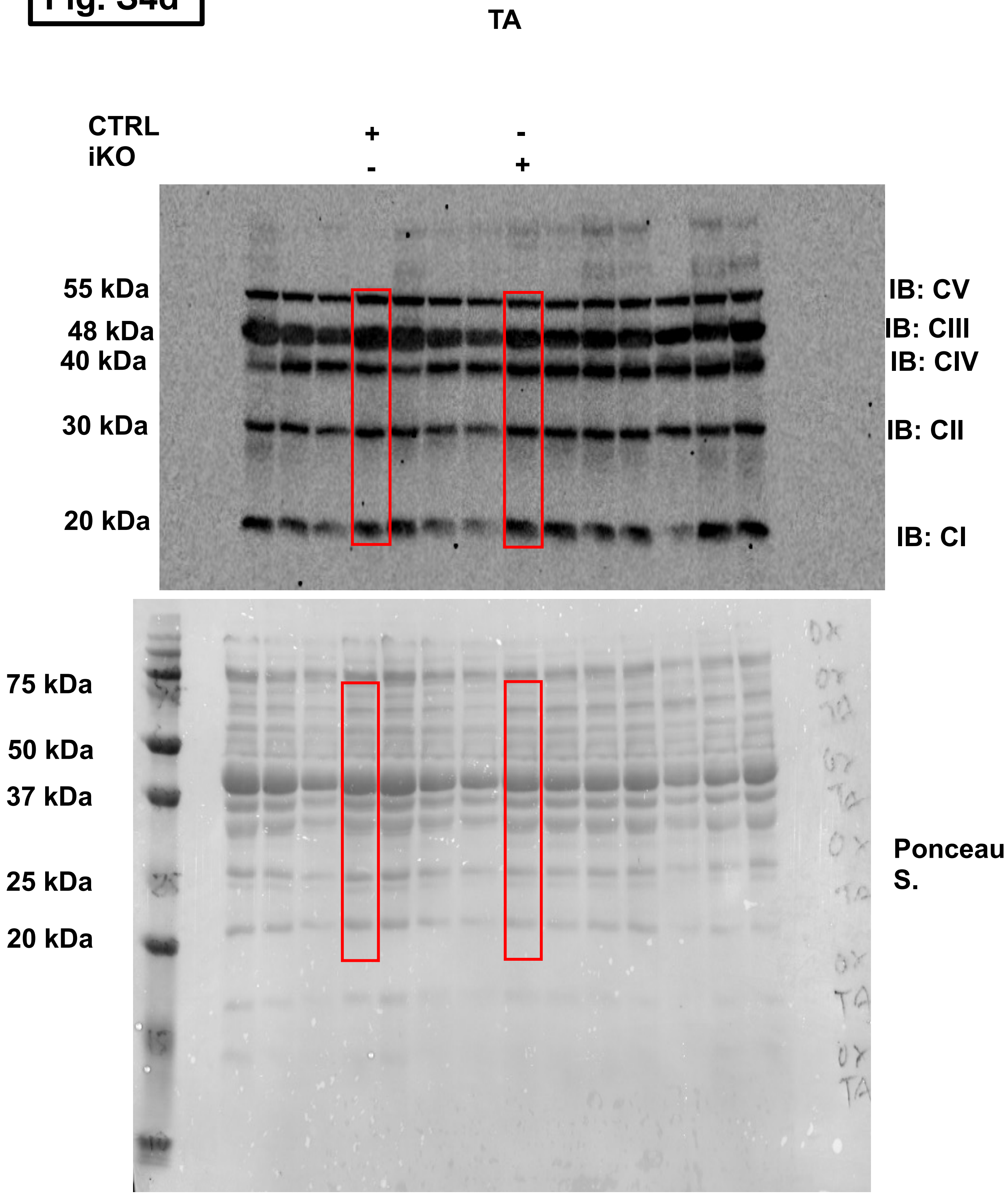
TA
CTRL + -
iKO - +
55 kDa
48 kDa
40 kDa
30 kDa
20 kDa
IB: CV
IB: CIII
IB: CIV
IB: CII
IB: CI
75 kDa
50 kDa
37 kDa
25 kDa
20 kDa
Ponceau S.

Fig. S5a

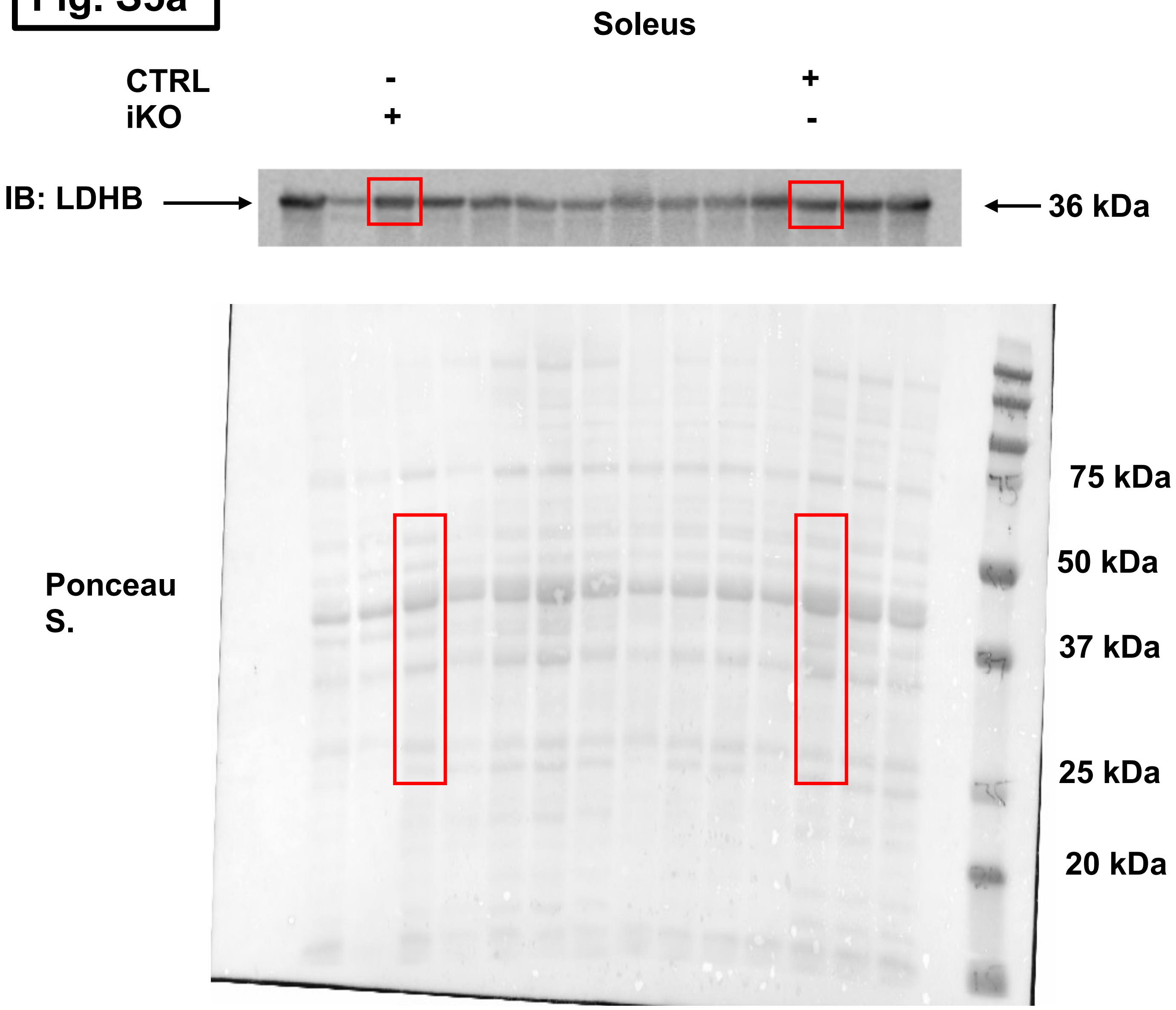
Soleus
CTRL - +
iKO + -
IB: LDHB
36 kDa
Ponceau S.
75 kDa
50 kDa
37 kDa
25 kDa
20 kDa

Fig. S5a

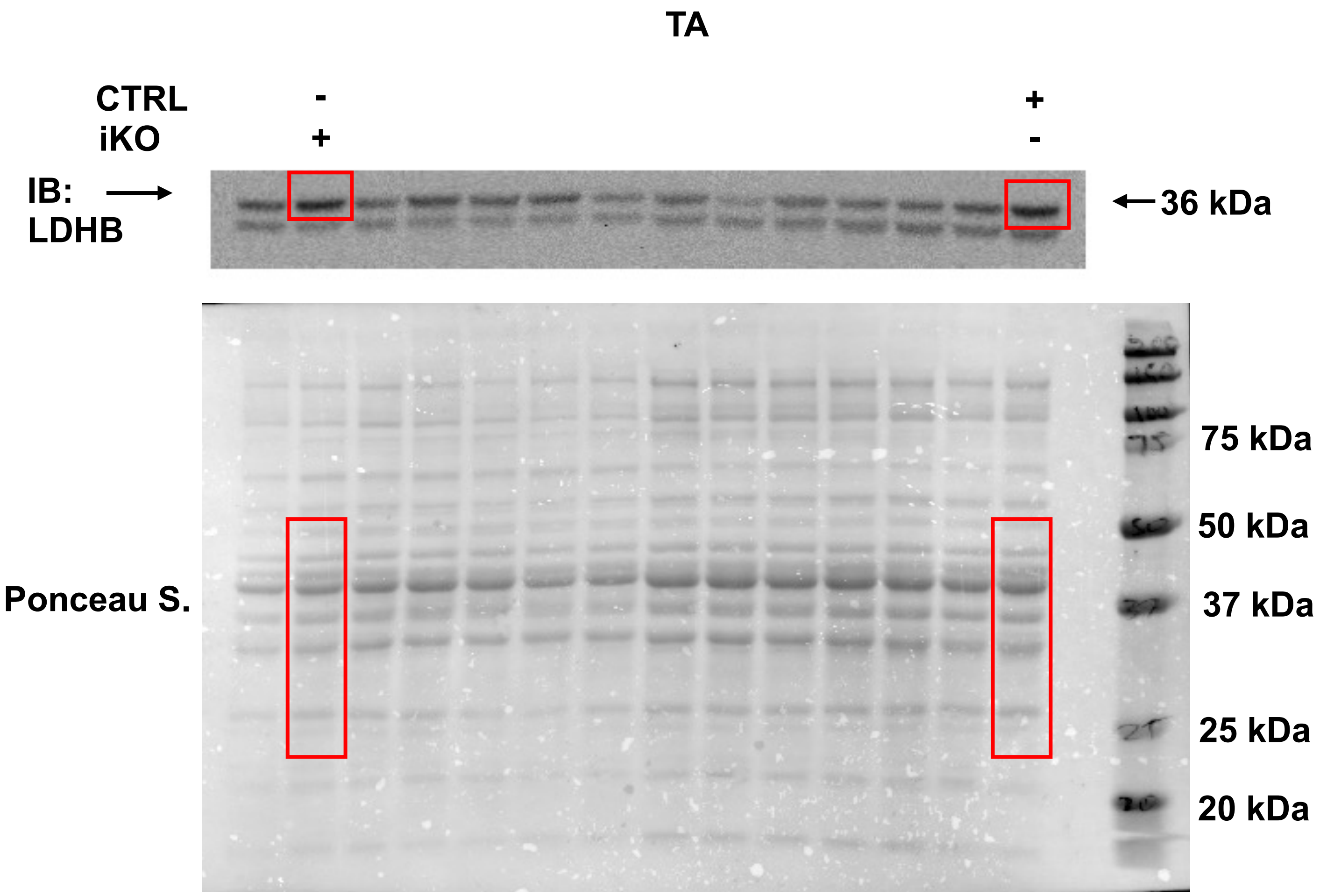
TA
CTRL - +
iKO + -
IB: LDHB
36 kDa
Ponceau S.
75 kDa
50 kDa
37 kDa
25 kDa
20 kDa

Fig. S5b

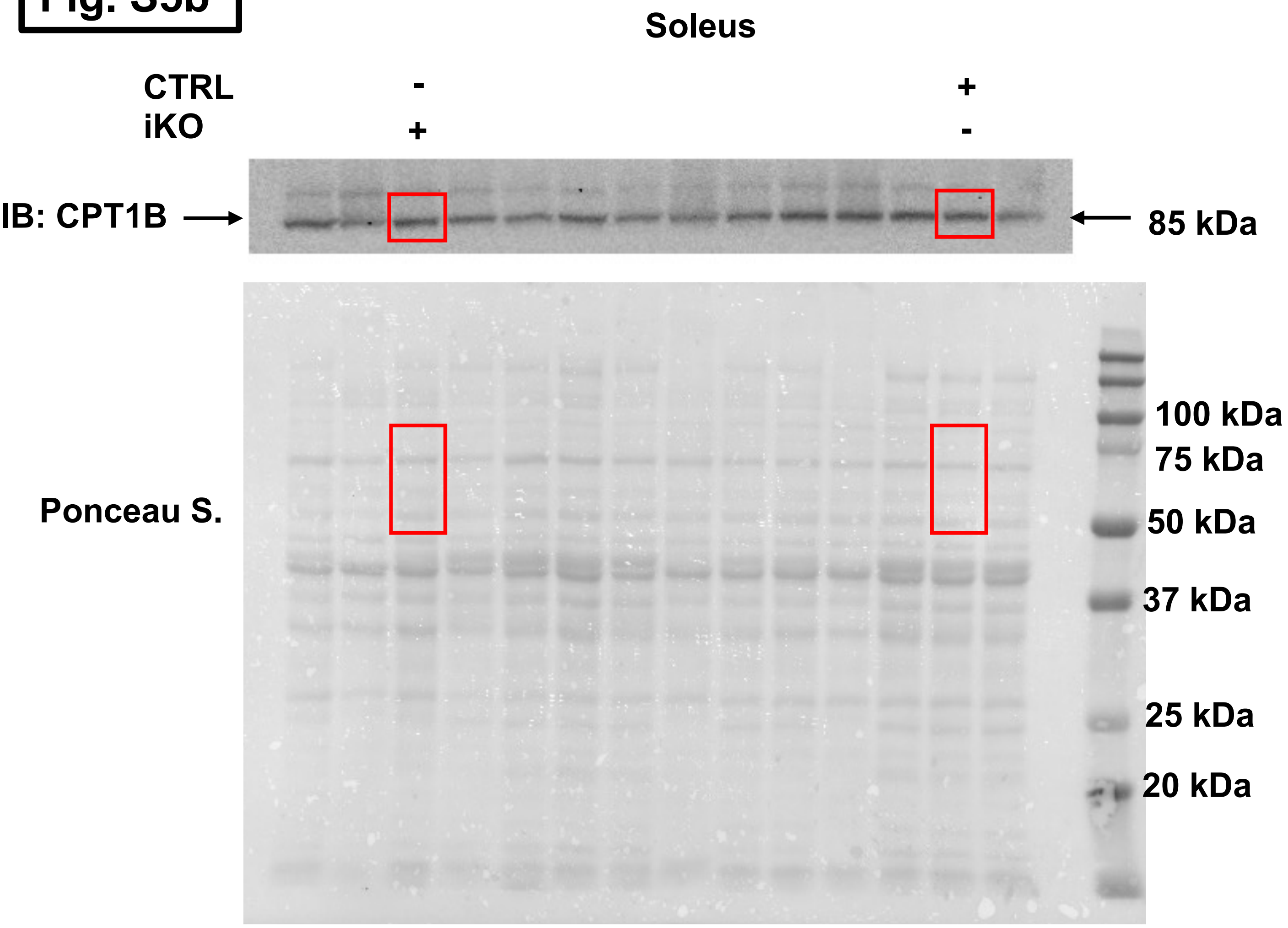
Soleus
CTRL - +
iKO + -
IB: CPT1B
85 kDa
Ponceau S.
100 kDa
75 kDa
50 kDa
37 kDa
25 kDa
20 kDa

Fig. S5b

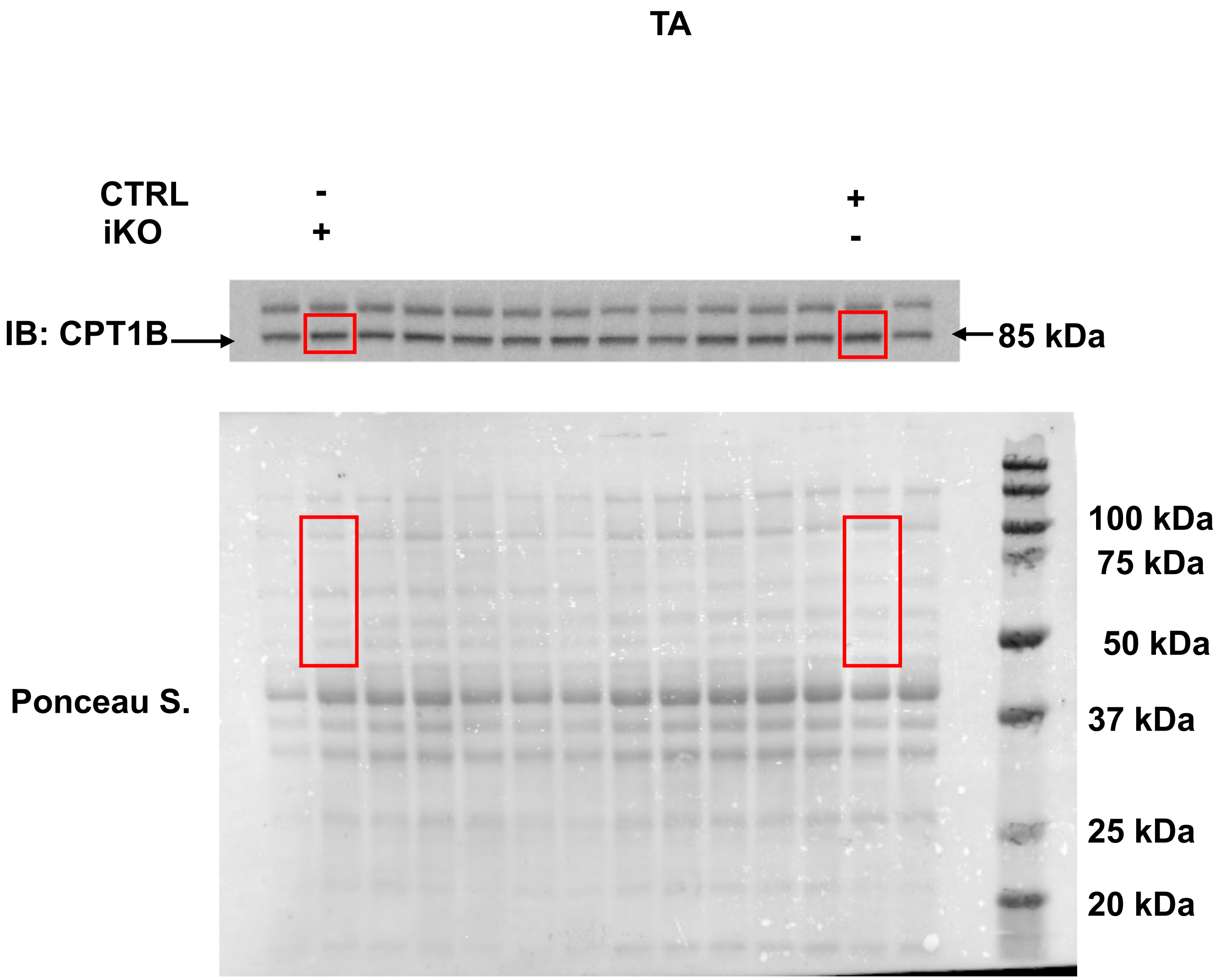
TA
CTRL
iKO
-
+
+
-
IB: CPT1B
85 kDa
Ponceau S.
100 kDa
75 kDa
50 kDa
37 kDa
25 kDa
20 kDa

Fig. S5C

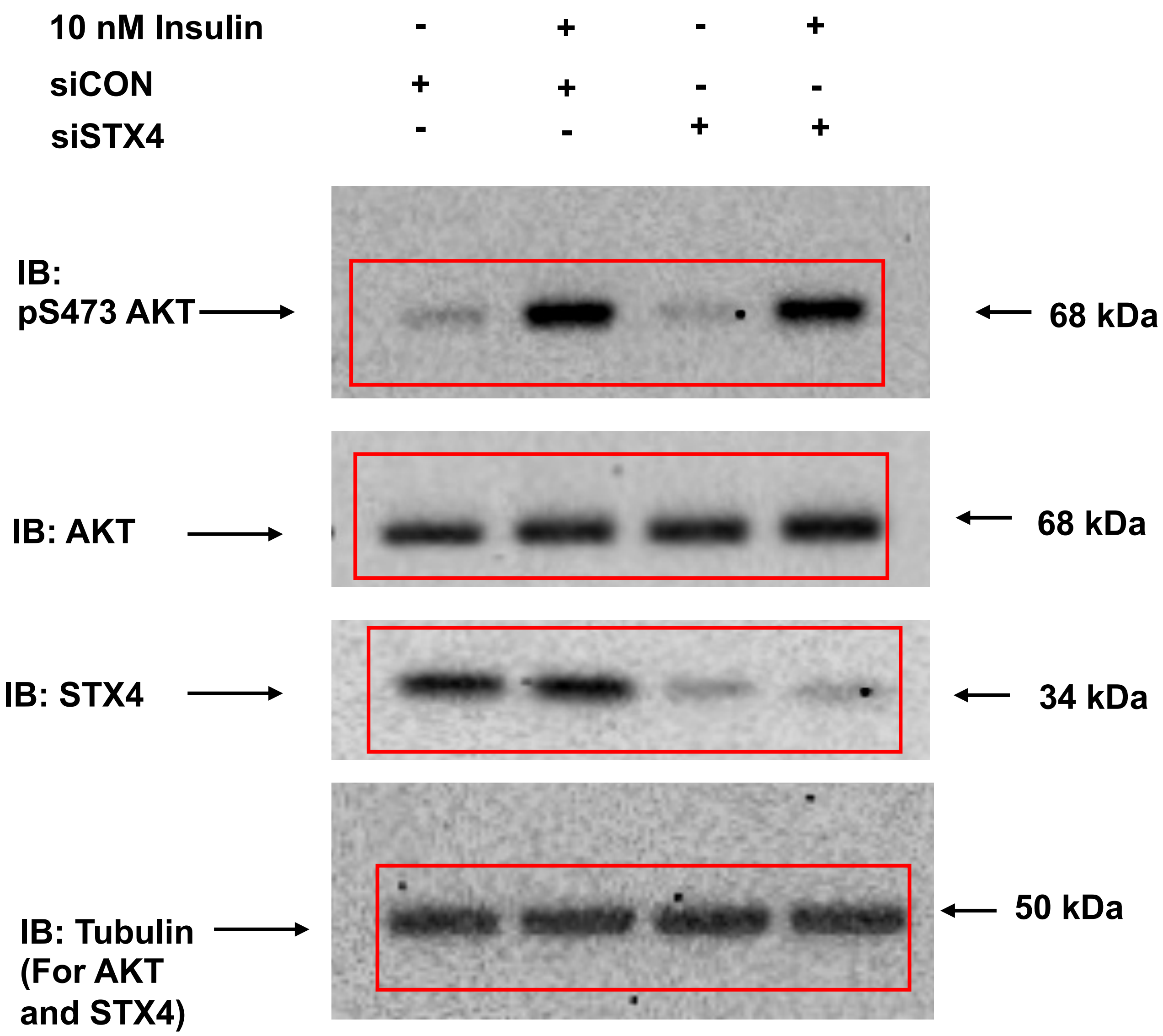

10 nM Insulin - + - +
siCON + + - -
siSTX4 - - + +
IB: pS473 AKT
68 kDa
IB: AKT
68 kDa
IB: STX4
34 kDa
IB: Tubulin (For AKT and STX4)
50 kDa
