# Supplementary material for: STX4 Is Indispensable for Mitochondrial Homeostasis in Skeletal Muscle
Source: J Cachexia Sarcopenia Muscle. 2025 Nov 10;16(6):e70113. doi: 10.1002/jcsm.70113 (PMC12602274; doi:10.1002/jcsm.70113)
Supplement: Supplementary file 2 — Table S1: Antibody list Table S2: Primer list Table S3: Tissue and body weights of chow‐fed CTRL and skmSTX4‐iKO (Dox‐induced) male mice. Table S4: Tissue and body weights of chow‐fed CTRL and skmSTX4‐iKO (Dox‐induced) female mice Figure S1: Male skmSTX4‐iKO mice present significantly reduced Stx4 mRNA levels and impaired glucose tolerance. Figure S2: Female skmSTX4‐iKO mice present reduced insulin tolerance and body weight. Figure S3: STX4 ablation has no impact on food/water consumption in male skmSTX4‐iKO mice. Figure S4: STX4 ablation has a heterogeneous impact on mitochondrial structure and electron transport chain complex abundance between oxidative‐rich soleus and glycolytic‐rich TA muscle in female skmSTX4 iKO mice. Figure S5: STX4 ablation does not impact markers for lactate conversion, fatty acid oxidation or canonical insulin signalling in skeletal muscle. Figure S6: STX4 ablation has no impact on mitochondrial biogenesis genes in the TA muscle in male skmSTX4‐iKO mice. [file JCSM-16-e70113-s001.pdf]

Supplemental Information

STX4 is indispensable for mitochondrial homeostasis in skeletal muscle

Joseph M. Hoolachan[1], Rekha Balakrishnan[1], Erika M. McCown[1], Karla E. Merz[1,2], Chunxue Zhou[1,3], Elizabeth Bloom-Saldana[1,4], Patrick T. Fueger[1,4], Angelica Hamilton[1,5], Tali Kiperman[6], Ke Ma[6], Eunjin Oh[1], Lei Jiang[7], Patrick Pirrotte[7,8], Orian Shirihai[9] & Debbie C. Thurmond[1].

1. Department of Molecular and Cellular Endocrinology, Arthur Riggs Diabetes and Metabolism Research Institute, City of Hope Beckman Research Institute, 1500 E. Duarte Road, Duarte, CA 91010, USA.
2. Amgen Inc, Thousand Oaks, CA, USA.
3. Sanegene Bio Inc. Suzhou, China.
4. Comprehensive Metabolic Phenotyping Core, Beckman Research Institute, 1500 E. Duarte Road, Duarte, CA, 91010, USA.
5. Clinical Research Division MC, City of Hope Beckman Research Institute, 1500 E. Duarte Road, Duarte, CA 91010, USA.
6. Department of Diabetes Complications and Metabolism, Arthur Riggs Diabetes and Metabolism Research Institute, City of Hope Beckman Research Institute, 1500 E. Duarte Road, Duarte, CA 91010, USA.
7. Integrated Mass Spectrometry Shared Resource, City of Hope Beckman Research Institute, 1500 E. Duarte Road, Duarte, CA 91010 USA.
8. Early Detection & Prevention Division, Translational Genomics Research Institute, 445 N. 5th Str, Phoenix, AZ, 85004, USA.
9. Department of Medicine, University of California Los Angeles, 100 Medical Plaza, Los Angeles, CA, 90095, USA.

Table of Contents:

Supplemental Table 1 ……………………………………………………………………………….....S2
Supplemental Table 2 ……………………………………………………………………………….S3
Supplemental Table 3 ……………………………………………………………………………..…S4
Supplemental Table 4 ……………………………………………………………………………..S5
Supplemental Figure 1 ……………………………………………………………………………S6
Supplemental Figure 2 …………………………………………………………………………....S7
Supplemental Figure 3 ……………………………………………………………………………S8
Supplemental Figure 4 ……………………………………………………………………..…..S9
Supplemental Figure 5 …………………………………………………………………………..S11
Supplemental Figure 6 …………………………………………………………………………..S13

Supplemental Table 1. Antibody List

Antibody	Vendor	Cat#	Size	Source	Dilution	RRID
STX4	In-house generated	N/A	34 kDa	Rabbit	1:5000	N/A
Tubulin	Sigma	T5168	50 kDa	Mouse	1:10,000	086M4773V
PGC1-a	Novus Biologics	NBP3-08971	91 kDa	Rabbit	1:1000	5500005011
NRF1	Abcam	Ab175932	68 kDa	Rabbit	1:1000	1008037-22
PINK1	Proteintech	23274-1-AP	65 kDa	Rabbit	1:1000	00146611
PARKIN	Biolegend	SIG-39530	50 kDa	Mouse	1:500	B322798
LDHB	Proteintech	14824-1-AP	35 kDa	Rabbit	1:1000	00154338
CPT1B	Proteintech	22170-1-AP	75 kDa	Rabbit	1:1000	0011708
AKT	Cell Signaling	2920S	68 kDa	Mouse	1:2000	8
pS473 AKT	Cell Signaling	4060S	68 kDa	Rabbit	1:2000	25
OXPHOS Cocktail (Rodent)	Abcam	Ab110413	CV – 55 kDa<br>CIII – 48 kDa<br>CIV – 40 kDa<br>CII – 30 kDa<br>CI – 20 kDa	Mouse	1:2000	2101039661

Supplemental Table 2. Primer List

Species	Gene	Forward Primer 5'-3'	Reverse Primer 5'-3'
Mouse	*Stx4*	CAG TAT CCG AGA GCT CCA TG	CCG AGC TCA GGA TGT TCT TCT C
	Ppargc1a	GAA TCA AGC CAC TAC AGA CAC CG	CAT CCC TCT TGA GCC TTT CGT G
	Nrf1	GGC AAC AGT AGC CAC ATT GGC T	GTC TGG ATG GTC ATT TCA TCA CCG C
	Tfam	GAG GCA AAG GAT GAT TCG GCT C	CGA ATC CTA TCA TCT TTA GCA AGC
	18S rDNA (Nuclear Gene)	TGG CTC ATT AAA TCA GTT ATG GT	GTC GGC ATG TAT TAG CTC TAG
	Cox1 (Mitochondrial Gene)	ACC ATC ATT TCT CCT TCT CCT A	TAG ATT TCC GGC TAG AGG TG
	Hprt	AAG CCT AAG ATG AGC GCA AG	TTA CTA GGC AGA TGG CCA CA

Rat	*Stx4*	CAC GAT CCG TGA ACT CCA TG	CTG CTG AGC TCA GAA TGT TCT TTT C
	Ppargc1a	TGA ACT ACG GGA TGG CAA C	AAG AGC AAG AAG GCG ACA C
	Nrf1	GGC GCA GCA CCT TTG GAG AAT GTG	CAT CGA TGG TGA GAG GGG GCA GTT C
	Tfam	ATC AAG ACT GTG CGT GCA TC	AGA ACT TCA CAA ACC CGC AC
	18S rDNA (Nuclear Gene)	TAG AGG GAC AAG TGG CGT TC	CGC TGA GCC AGT CAG TGT
	Mt-cyb (Mitochondrial Gene)	TCC ACT TCA TCC TCC CAT TC	CTG CGT CGG AGT TTA ATC CT
	Hprt	GGT CCA TTC CTA TGA CTG TAG ATT TT	CAA TCA AGA CGT TCT TTC CAG TT

Supplemental Table 3. Tissue and body weights of chow-fed CTRL and skmSTX4-iKO (Dox-induced) male mice.

	CTRL (n = 13)	skmSTX4-iKO (n = 11)	p-value (two-tailed t-test)
Bodyweight (g)	27.68 ± 1.21	24.05 ± 1.20	<0.0001 (****)
Tissue (% Body weight)			
Kidney	1.29 ± 0.03	1.54 ± 0.07	0.0057 (**)
Heart	0.71 ± 0.03	0.82 ± 0.07	0.1406
Spleen	0.35 ± 0.03	0.43 ± 0.05	0.2066
Pancreas	0.80 ± 0.03	0.91 ± 0.04	0.056
Fat	1.93 ± 0.17	1.94 ± 0.18	0.5067
Liver	5.19 ± 0.70	5.98 ± 0.12	<0.0001 (****)
Lungs	0.87 ± 0.10	0.88 ± 0.05	0.2486
Brain	1.51 ± 0.05	1.75 ± 0.04	0.0032 (**)
Quadriceps Muscle	1.43 ± 0.13	1.00 ± 0.80	<0.0001 (****)
Gastrocnemius Muscle	1.14 ± 0.15	0.76 ± 0.15	<0.0001 (****)
Tibialis Muscle	0.72 ± 0.16	0.75 ± 0.20	0.1858
Soleus Muscle	0.26 ± 0.10	0.35 ± 0.19	0.3960

Data represents the average ± SD of 20-week-old male mice. Significant differences detected using unpaired two-tailed t-test.

Supplemental Table 4. Tissue and body weights of chow-fed CTRL and skmSTX4-iKO (Dox-induced) female mice.

	CTRL (n = 8)	skmSTX4-iKO (n = 8)	p-value (two-tailed t-test)
Bodyweight (g)	21.75 ± 2.55	19.62 ± 1.85	0.0384 (*)
Tissue (% Body weight)			
Kidney	1.14 ± 0.10	1.21 ± 0.14	0.3905
Heart	0.65 ± 0.09	0.62 ± 0.18	0.7278
Spleen	0.26 ± 0.07	0.38 ± 0.10	0.0549
Pancreas	0.90 ± 0.21	0.99 ± 0.18	0.4569
Fat	1.85 ± 1.21	0.99 ± 0.36	0.0995
Liver	5.07 ± 0.62	5.71 ± 0.70	0.1396
Lungs	0.71 ± 0.10	0.76 ± 0.05	0.4403
Brain	1.79 ± 0.21	2.10 ± 0.12	0.0153 (*)
Quadriceps Muscle	1.21 ± 0.17	0.73 ± 0.14	<0.0001 (****)
Gastrocnemius Muscle	0.96 ± 0.15	0.59 ± 0.13	<0.0001 (****)
Tibialis Muscle	0.45 ± 0.05	0.39 ± 0.09	0.0998
Soleus Muscle	0.05 ± 0.03	0.06 ± 0.05	0.6763

Data represents the average ± SD of 20-week-old female mice. Significant differences detected using unpaired two-tailed t-test.

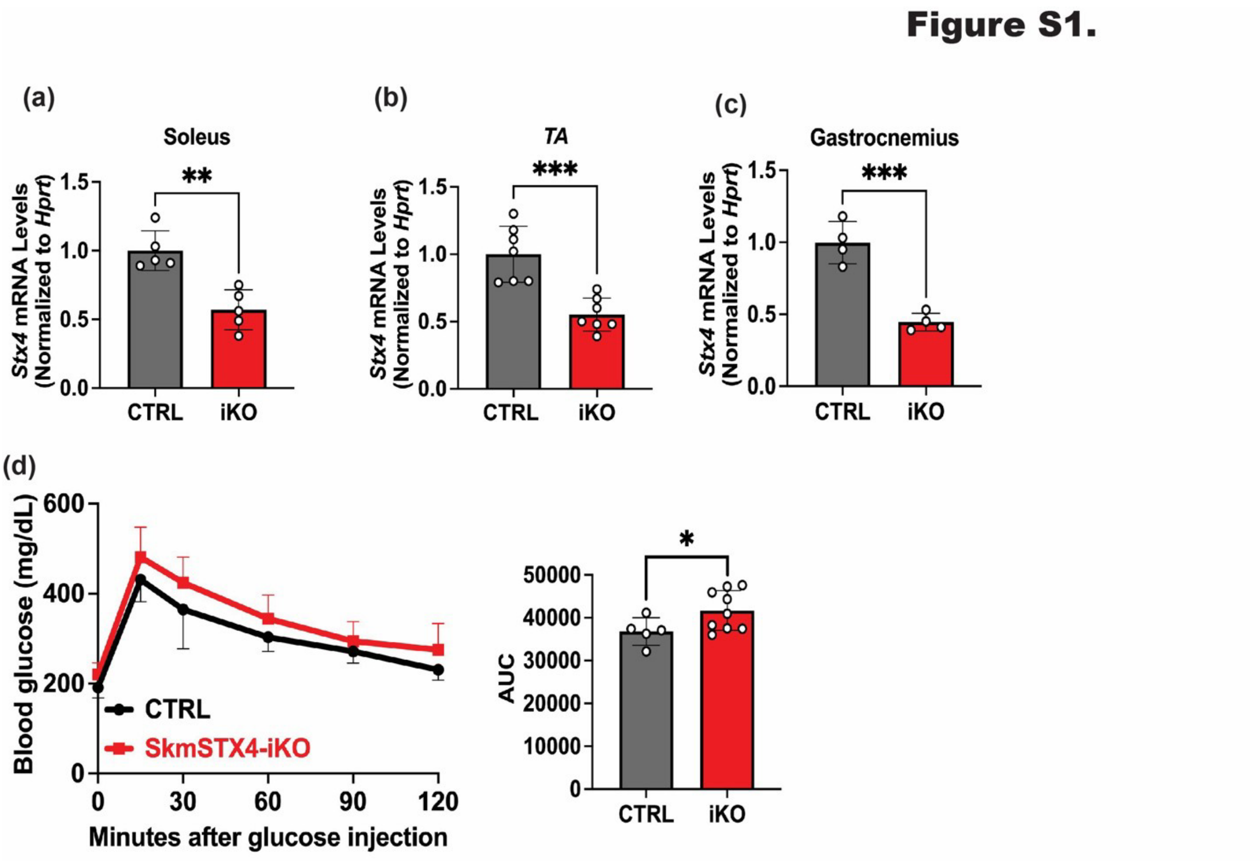

Figure S1. Male skmSTX4-iKO mice present significantly reduced *Stx4* mRNA levels and impaired glucose tolerance.

Quantification of mRNA levels for *Stx4* normalized against *Hprt* reference gene between control (CTRL, Grey) and skmSTX4-iKO (iKO, Red) 20-week-old male mice across (**a**) soleus, (**b**) Tibialis anterior (TA), and (**c**) gastrocnemius (GAS) muscle (n=4-6 mice/group). (**d**) Intraperitoneal glucose tolerance test (IPGTT) between control (CTRL, Black/Grey) and skmSTX4-iKO (iKO, Red) 20-week-old male mice with area under the curve (AUC) bar chart (n=5-9 mice/group). Data in bar and line graphs shown as mean ± SD. Statistical significance was determined by unpaired two tailed Student's t test (**a, b, c** and **d** (AUC)) and multiple t-test model (**d**). *p<0.05, **p<0.01, ***p<0.001.

Supplemental Figure 2.

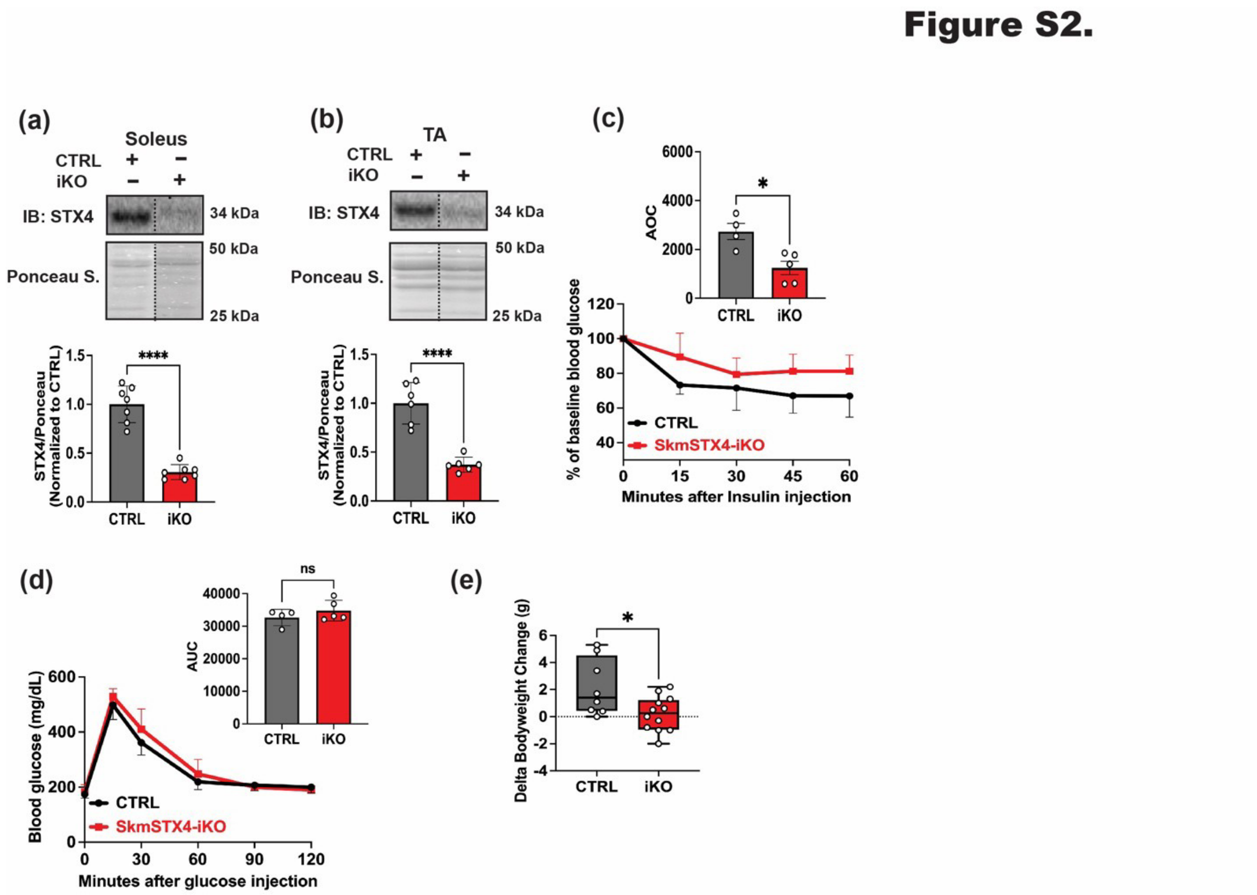

Figure S2. Female skmSTX4-iKO mice present reduced insulin tolerance and bodyweight.

Representative immunoblot (top) and quantification (bottom) of STX4 protein abundance across skeletal muscle depots (**a**) soleus and (**b**) Tibialis anterior from control (CTRL, grey) and skmSTX4-iKO (iKO, red) 20-week-old female mice normalized against total protein lysate stained with ponceau. (**c**) Intraperitoneal insulin tolerance test (IPITT) (bottom) of CTRL (black/grey) and skmSTX4-iKO (iKO, red) female 20-week-old mice with area over the curve (AOC) bar chart. (**d**) Intraperitoneal glucose tolerance test (IPGTT) between CTRL (Black/Grey) and skmSTX4- iKO (Red) 20-week-old female mice with area under the curve (AUC) bar chart. **(e)** Delta bodyweight change of initial weight to final weight of control (CTRL, grey) and skmSTX4-iKO (iKO, red) 20-week-old female mice. Data represents n = 4-12 mice per group in bar and line graphs shown as mean ± SD. Statistical significance was determined by unpaired two tailed Student's t test (**a, b, c** (AOC), **d** (AUC), **e**) and multiple t-test model (**c, d**). *p<0.05,

****p<0.0001, ns = not significant. Vertical dashed lines indicate the splicing of lanes within the same gel exposure. IB: Immunoblot, Ponceau S.: Ponceau stain.

Supplemental Figure 3.

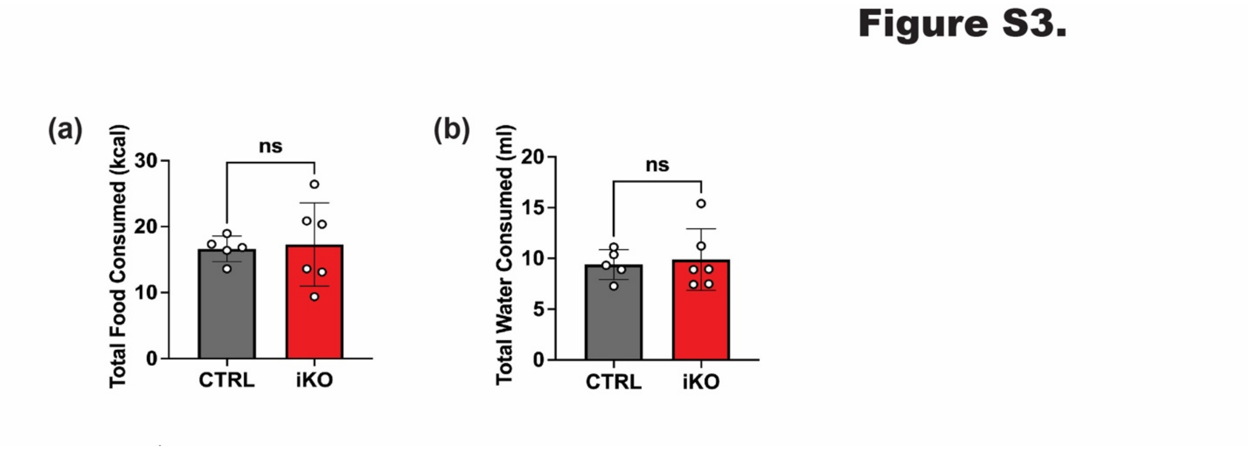

Figure S3. STX4 ablation has no impact on food/water consumption in male skmSTX4-iKO mice.

Metabolic caging analyses of the 26-week-old control (CTRL, black) and skmSTX4-iKO (iKO, red) male mice 72 hours period (n=5-6 mice/group) for (**a**) Total food consumed and (**b**) Total water consumed. Bar graph represents mean ± SD, unpaired two-tailed Student's *t*-test, ns = not significant.

Supplemental Figure 4.

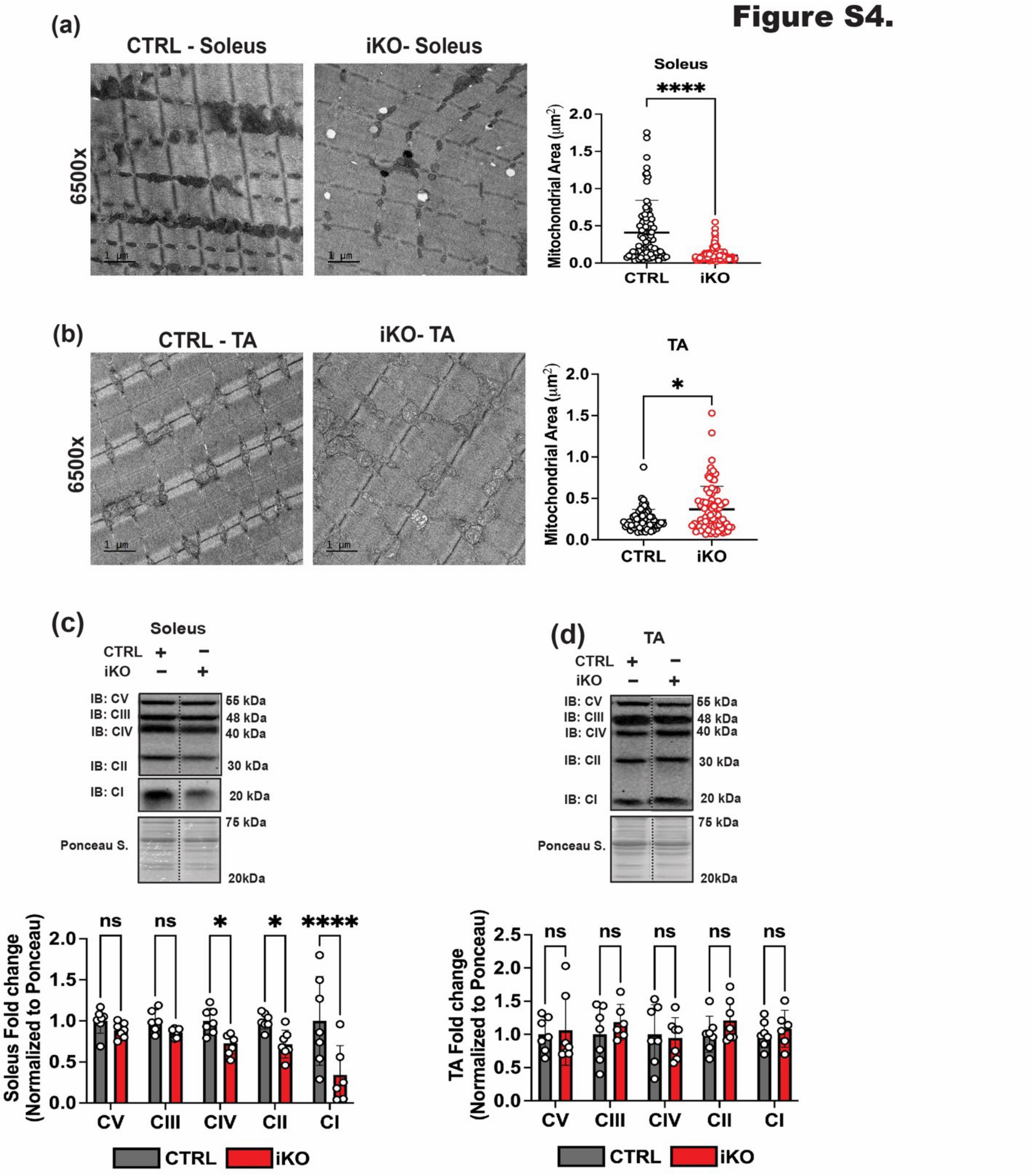

Figure S4. STX4 ablation has a heterogenous impact on mitochondrial structure and electron transport chain complex abundance between oxidative rich soleus and glycolytic rich TA muscle in female skmSTX4-iKO mice. Representative transmission electron microscopy images and mitochondrial area (μm^2) quantification of mitochondria from **(a)** Soleus (n = 96-202 total mitochondria) and **(b)** Tibialis anterior (TA) muscle (n=63-85 total

mitochondria) from control (CTRL, Grey) and skmSTX4-iKO (iKO, Red) 20-week-old female mice. Black bar = 1 μm (6500X). Representative immunoblot (top) and quantification (bottom) of total electron transport chain (ETC) complexes V (CV), III (CIII), IV (CIV), II (CII) and I (CI) normalized against total protein lysate stained with ponceau in **(c)** soleus and **(d)** TA muscle from control (CTRL, Grey) and skmSTX4-iKO (iKO, Red) 20-week-old female mice (n=7 mice/group). Data in bar graphs shown as mean ± SD. Statistical significance was determined by unpaired two tailed Student's *t* test (**a**), Mann-Whitney U test (**b**), and two-way ANOVA with uncorrected Fisher's LSD test (**c, d**). *p<0.05, ***p<0.001, ****p<0.0001, ns = not significant. Vertical dashed lines indicate the splicing of lanes within the same gel exposure. IB: Immunoblot, Ponceau S.: Ponceau stain.

Supplemental Figure 5.

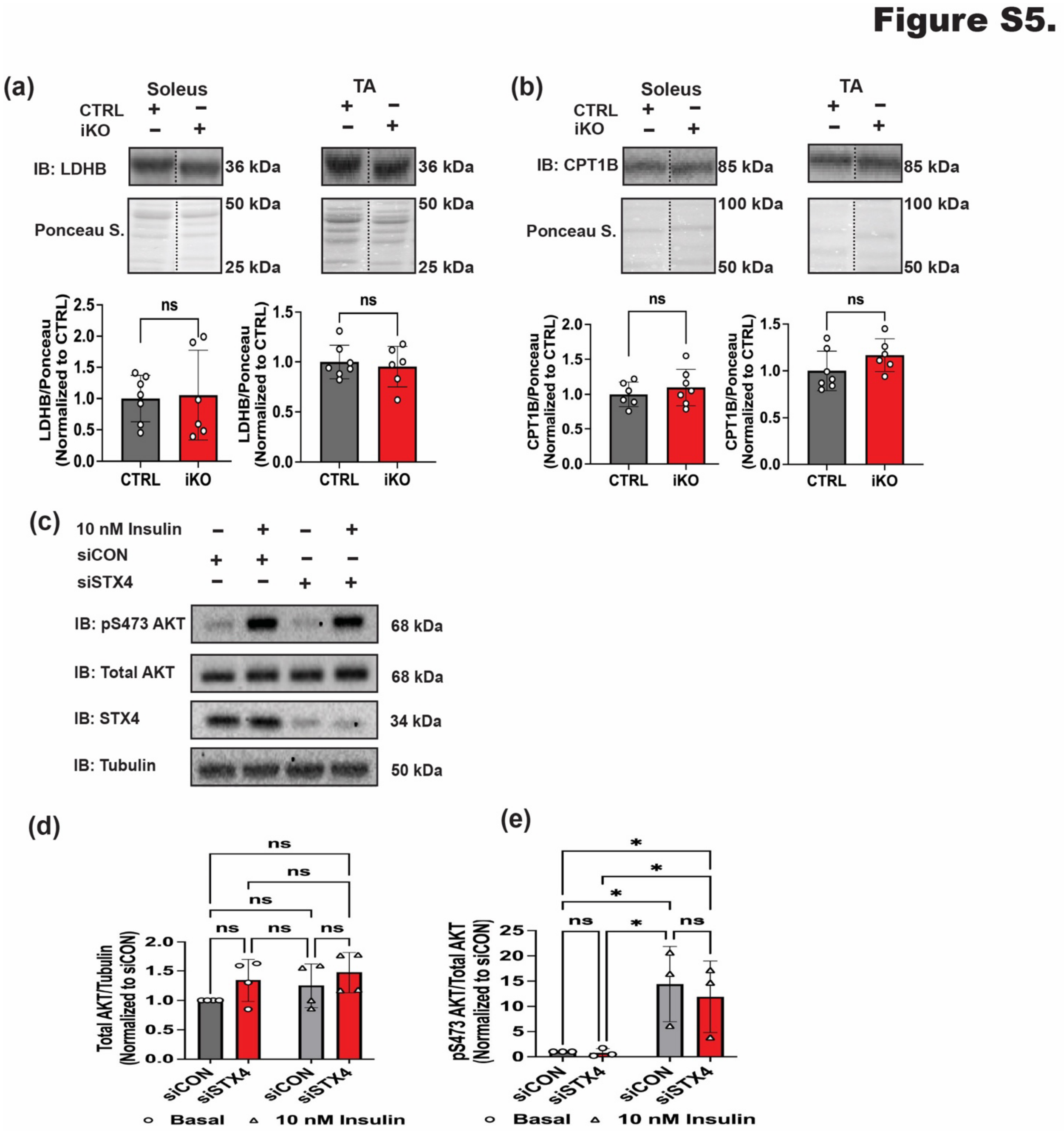

Figure S5. STX4 ablation does not impact markers for lactate conversion, fatty acid oxidation or canonical insulin signaling in skeletal muscle. Representative immunoblot (top) and quantification (bottom) of (**a**) LDHB and (**b**) CPT1B normalized against total protein lysate with ponceau in soleus and Tibialis anterior (TA) muscle from control (CTRL, Grey) and skmSTX4-iKO (iKO, Red) 20-week-old male mice (n=7 mice/group). (**c**) Representative immunoblot of pS473 AKT, total AKT, STX4 and Tubulin with quantification for (**d**) total AKT and (**e**) pS473 AKT normalized against Tubulin in siCON (Grey) and siSTX4 (Red) L6.GLUT4myc myotubes with or without 10 nM

insulin stimulation (n=3-4 independent passages). Data in bar graphs shown as mean ± SD. Statistical significance was determined by unpaired two tailed Student's *t* test (**a, b**) and two-way ANOVA with uncorrected Fisher's LSD test (**c, d**). *p<0.05, ns = not significant. Vertical dashed lines indicate the splicing of lanes within the same gel exposure. IB: Immunoblot, Ponceau S.: Ponceau stain.

Supplemental Figure 6.

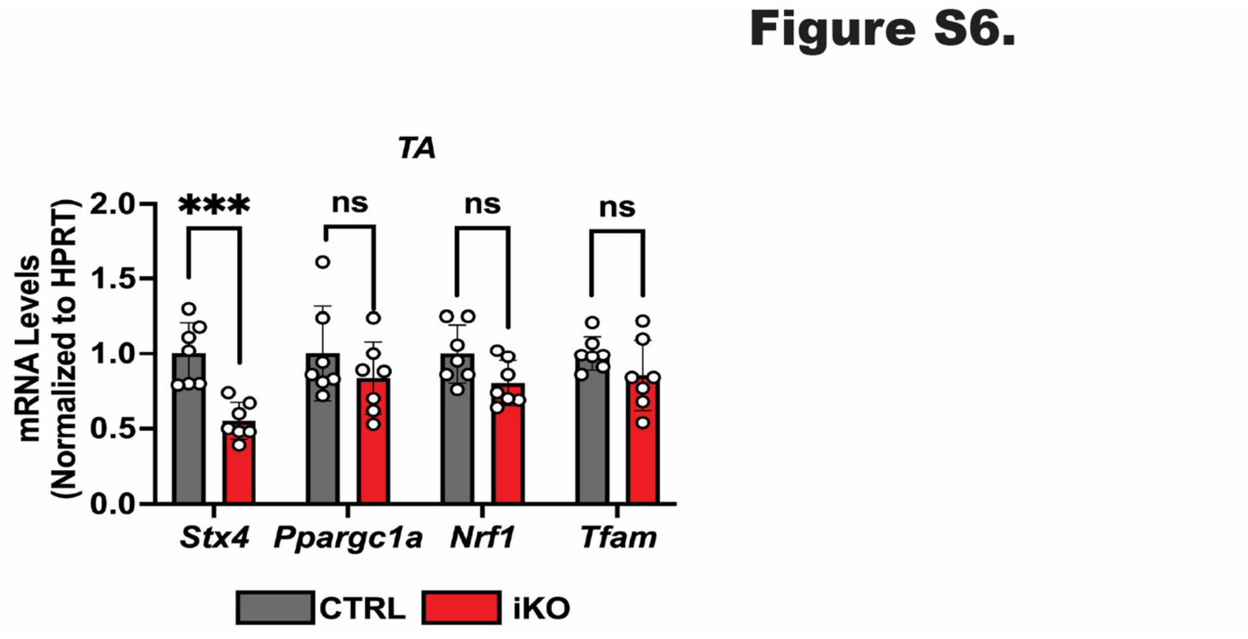

Figure S6. STX4 ablation has no impact on mitochondrial biogenesis genes in the TA muscle in male skmSTX4-iKO mice. Quantification of mRNA levels via qPCR of *Stx4*, *Ppargc1a* (Pgc1-α), *Nrf1* and *Tfam* normalized against housekeeping gene *Hprt* in the Tibialis anterior (TA) muscle of 20-week-old CTRL (Grey) and skmSTX4-iKO (Red) male mice. Data represents n = 7 mice per group in bar graphs shown as mean ± SD. Statistical significance was determined two-way ANOVA with uncorrected Fisher's LSD test, ***p<0.001, ns = not significant.
