# Supplementary material for: STX4 Is Indispensable for Mitochondrial Homeostasis in Skeletal Muscle
Source: J Cachexia Sarcopenia Muscle. 2025 Nov 10;16(6):e70113. doi: 10.1002/jcsm.70113 (PMC12602274; doi:10.1002/jcsm.70113)
Supplement: Supplementary file 3 — Data S2: Supplementary references. [file JCSM-16-e70113-s002.docx]

**Supplementary References**

1. Ye S, Karim ZA, Al Hawas R, Pessin JE, Filipovich AH, Whiteheart SW. Syntaxin-11, but not syntaxin-2 or syntaxin-4, is required for platelet secretion. Blood 2012;120:2484–92. <https://doi.org/10.1182/blood-2012-05-430603>.
2. Gao H, Xiong X, Lin Y, Chatterjee S, Ma K. The clock regulator Bmal1 protects against muscular dystrophy. Experimental Cell Research 2020; 397 (1) 112348. <https://doi.org/10.1016/j.yexcr.2020.112348>
3. Jun L, Knight E, Broderick TL, Al-Nakkash L, Tobin B, Geetha T, Babu JR. Moderate-intensity exercise enhances mitochondrial biogenesis markers in the skeletal muscle of a mouse model affected by diet-induced obesity. Nutrients 2024; 16: 1836. <https://doi.org/10.3390/nu16121836>
4. Ashrafi G, Schwarz TL. The pathways of mitophagy for quality control and clearance of mitochondria. Cell Death Differ 2013;20:31–42. <https://doi.org/10.1038/cdd.2012.81>.
5. Sun N, Malide D, Liu J, Rovira II, Combs CA, Finkel T. A fluorescence-based imaging method to measure in vitro and in vivo mitophagy using mt-Keima. Nat Protoc 2017;12:1576–87. <https://doi.org/10.1038/nprot.2017.060>.
6. Tang X, Miao Y, Luo Y, Sriram K, Qi Z, Lin F-M, et al. Suppression of endothelial AGO1 promotes adipose tissue browning and improves metabolic dysfunction. Circulation 2020;142:365–79. <https://doi.org/10.1161/CIRCULATIONAHA.119.041231>.
7. Singh A, D’Amico D, Andreux PA. Fouassier AM, Blanco-Bose W, Evans M, Aebischer P, Auwerx J, Rinsch C. Urolithin A improves muscle strength, exercise performance, and biomarkers of mitochondrial health in a randomized trial in middle-aged adults. Cells Reports Medicine 2022; 3(5): 10063.
8. Qiu S, Cai X, Yuan Y, Xie B, Sun Z, Wang D, Wu T. Muscle strength and prediabetes progression and regression in middle-aged and older adults: a prospective cohort study. Journal of Cachexia, Sarcopenia and Muscle 2022. 13(2): 909-918
9. Walsh MA, Zhang Q, Musci RV, Hamilton KL. The combination of NRF1 and Nrf2 activators in myoblasts stimulate mechanisms of proteostasis without changes in mitochondrial respiration. Redox in Muscle Physiology, Exercise, and Sport 2022;1:100001. https://doi.org/10.1016/j.rimpes.2022.100001.
10. Chepelev NL, Bennitz JD, Huang T, McBride S, Willmore WG. The Nrf1 CNC-bZIP protein is regulated by the proteasome and activated by hypoxia. PLoS One 2011;6:e29167. <https://doi.org/10.1371/journal.pone.0029167>.
